# Can Potentiometric Titration Be Almost as Accurate as ^1^H NMR to Determine the Degree of Deacetylation of Modified Chitosan?

**DOI:** 10.3390/molecules31213892

**Published:** 2026-10-28

**Authors:** Sébastien Cardinal, Tommy Fraser, Sarah Kefil

**Affiliations:** 1 Département de Biologie, Chimie et Géographie, Université du Québec à Rimouski, Rimouski, QC G5L 3A1, Canada; tommy.fraser.1@ulaval.ca (T.F.); sarah.kefil@uqar.ca (S.K.); 2 Département de Chimie, Centre d’Études Nordiques and PROTEO, Université Laval, Quebec City, QC G1V 0A6, Canada

**Keywords:** chitosan, degree of deacetylation (DDA), determination of DDA of modified chitosan, ^1^H NMR, potentiometric titration, equation, naphthalene–chitosan derivatives

## Abstract

Chitosan is a linear biopolymer composed of randomly distributed D-glucosamine and *N*-acetylglucosamine repeating units. Various nucleophilic functionalities within its structure make chitosan easy to modify by chemical synthesis to generate new tailor-made chitosan derivatives with specific properties. One parameter that heavily influences these properties is the degree of deacetylation (DDA), which is the percentage of D-glucosamine units (of the total repeating units) in the polymer chains. Various methods to determine the DDA of unmodified or modified chitosans exist, including ^1^H NMR and potentiometric titration. ^1^H NMR is generally considered to be the most accurate method, but is not always readily available. Potentiometric titration relies on more accessible equipment but offers less accurate results. This is particularly the case with heavily modified chitosan derivatives, since the equation generally used to calculate the DDA after the titration does not consider the molecular weight of the modified repeating units that are part of the polymeric chain. To circumvent this limitation, we recently reported a new equation that is better suited to determine the DDA of chitosan derivatives with three types of repeating units. In our previous work, we tested this equation by preparing naphthalene–chitosan derivatives and subjecting them to potentiometric titration. We showed that this new equation gives higher DDA values than the traditional equation, suggesting that classic potentiometric titration underestimates the DDA of modified chitosans. In this paper, we used ^1^H NMR to calculate the DDA of a series of naphthalene–chitosan derivatives and compared those new DDA values with the results previously obtained by potentiometric titration. As we show here, for our series of naphthalene–chitosan derivatives, our new equation calculates the DDA values with potentiometric titration more accurately (i.e., more in agreement with the NMR ^1^H result) than the previous equation by up to 5 DDA units (%).

## 1. Introduction

Chitosan is the product of partial deacetylation of chitin. The structure of this biopolymer is composed of randomly distributed D-glucosamine and *N*-acetylglucosamine units chained together by a β-1–4 glycosidic linkage [1,2]. Chitosan has received considerable attention in recent decades, due to its antimicrobial properties and its biodegradability. It is useful in a wide range of applications, including pharmaceuticals, wastewater treatment, agriculture, textiles, and cosmetics [3,4,5].

Depending on the natural or commercial source, chitosans can exist in various structural forms and thus exhibit various properties. Two main structural parameters are used to characterize a chitosan sample: the length of the linear polymeric chain (most expressed in terms of molecular weight of the polymeric chain) and the degree of deacetylation (DDA). The DDA is defined as the percentage of D-glucosamine units (of the total repeating units) in the polymer chain [6].

Due to the presence of free amines, chitosans can be subjected to various chemical reactions (e.g., cross-linking, imination, acylation, alkylation) to produce new synthetic analogues with different properties. One great advantage of this chemical reactivity is that it allows for the preparation of tailor-made chitosan derivatives with specific properties [7,8,9,10].

As for unmodified chitosan, one of the key factors that determines the chemical, physical, and biological properties of a modified chitosan is the ratio of D-glucosamine repeating units remaining in its polymer chains (i.e., its DDA). Consequently, the accurate measurement of the DDA of modified chitosan can often be crucial. Furthermore, monitoring the DDA of a chitosan sample before and after its chemical modification can be a simple and straightforward way to calculate the level of incorporation of the new chemical moiety on the polymer chains [6].

Numerous methods are available to determine the DDA of unmodified or modified chitosans. These include infrared spectroscopy [11], potentiometric titration [12,13,14,15], X-ray diffraction [16], ^1^H and ^13^C NMR [17,18,19], and ultraviolet spectroscopy [20]. To date, ^1^H NMR is considered the method of choice for determining the DDA of a chitosan sample, as it is generally viewed as the most accurate [21,22]. However, not all research teams have access to a costly and high-maintenance high-field NMR facility. Also, as the solubilization of modified chitosan derivatives can often be challenging, the use of solid-state NMR—an even less accessible technique—may be needed.

Alternatively, potentiometric titration is a simple and much more accessible technique to determine the DDA of a chitosan sample. This method, which relies on readily accessible reagents and equipment [12,13,14], was first introduced in 1968 by Broussignac [12] and has since been widely used to evaluate the DDA of modified and unmodified chitosans [23,24]. However, this method is generally considered less accurate than ^1^H NMR. This is particularly the case with heavily modified chitosan derivatives [25], partly because the classic equation used to convert the raw experimental data obtained by titration (a volume of NaOH) into the DDA does not consider the introduction of a new repeating unit (with its own different molecular weight) induced by the chemical modification.

To circumvent this limitation, we recently introduced a new equation especially suited to determine the DDA of modified chitosan [26]. In our previous work, we demonstrated, using a series of naphthalene-chitosan derivatives, that this new equation allowed us to obtain significantly higher values of DDA when compared with the classical equations. Since our equation considers a more accurate portrait of the chitosan derivative structure, it likely also offers more accuracy for DDA values by potentiometric titration [26].

Herein, we will compare the DDA values obtained with our equation with the results obtained by ^1^H NMR to confirm this assumption. We aim to answer this simple question: can potentiometric titration be almost as accurate as ^1^H NMR to determine the degree of deacetylation of modified chitosan?

## 2. Context

The principle behind the determination of DDA with potentiometric titration is rather simple. The chitosan derivative is first dissolved in an aqueous HCl solution to ensure that all the amino groups (NH_2_) are protonated to their ammonium form (NH_3_^+^). Then, the resulting mixture is titrated with aqueous NaOH while the pH value is precisely monitored. During titration, two inflection points (*d*^2^*pH*/*dV*^2^ = 0) are observed, each associated with an added volume of NaOH (*V*_1_ and *V*_2_). *V*_1_ is the volume necessary to neutralize the excess HCl used to dissolve the chitosan derivative, while *V*_2_ is the total volume of NaOH added while all the ammonium functionalities (NH_3_^+^) on the chitosan return to their amino form (NH_2_). The difference between *V*_2_ and *V*_1_ (defined as Δ*V*) represents the volume of NaOH used to neutralize the ammonium functions of the chitosan. This difference (Δ*V*) is the key experimental data obtained from the potentiometric titration. Examples of titration curves and the accompanying detailed procedure can be found in our previous studies [6,26].

Once Δ*V* is obtained by potentiometric titration, the value of *DDA* can be obtained using an appropriate equation involving a series of other variables. The equation most commonly used is still the one introduced by Broussignac in his seminal work [12]. It is labelled here as Equation (1):

(1) $$DDA\;\left(\%\right)=\;100\frac{\left(203.19\;g/\mathrm{mol}\cdot ∆V\cdot \left[\mathrm{NaOH}\right]\right)}{m+\left(42.03\;g/\mathrm{mol}\cdot ∆V\cdot \left[\mathrm{NaOH}\right]\right)}\;$$

for which [NaOH] is the concentration (mol/L) of the sodium hydroxide solution used for titration, Δ*V* (*V*_2_ − *V*_1_) is the volume (L) of this NaOH solution required to neutralize the ammonium functionalities, 203.19 (g/mol) is the molecular weight of the *N*-acetylglucosamine repeating unit, 42.03 (g/mol) is the difference between the molecular weight of the *N*-acetylglucosamine repeating unit and that of the D-glucosamine repeating unit, and *m* is the mass (g) of the sample before titration.

This equation allows us to obtain a DDA value as accurate as possible for the titration of unmodified chitosan, since it considers the respective molecular weight of the two repeating units that make up its structure.

However, this equation becomes less and less accurate as a chitosan derivative gets modified. Indeed, the chemical modification of chitosan usually involves the introduction of a third repeating unit (with its distinct molecular weight) to the polymeric chain. In almost all cases, this new repeating unit has a higher molecular weight than the glucosamine and the *N*-acetylglucosamine repeating units. Neglecting the presence of that third repeating unit with Equation (1) obviously leads to a loss of accuracy in the determination of DDA by potentiometric titration.

To overcome this problem, we recently reported a new equation designed for the determination of the DDA of modified chitosan having three different repeating units in their polymeric structure [26]. The generic form of this equation is labeled here as Equation (2):

(2) $$DDA\;\left(\%\right)\;=100\frac{\left(∆V\cdot \left[\mathrm{NaOH}\right]\right)\left(\left(203.19\;g/\mathrm{mol}\cdot DA\right)+\left(M_{mod-mono}\cdot DDA_{SM}\right)\right)}{m_{chit}+\left(∆V\cdot \left[\mathrm{NaOH}\right]\cdot ∆M\right)}$$

for which Δ*V* is the difference between the two inflection points (L), [NaOH] is the concentration of the sodium hydroxide solution used for titration (mol/L), 203.19 g/mol is the molecular weight of the *N*-acetylglucosamine repeating unit, *DDA_SM_* is the degree of deacetylation of the chitosan starting material used for modification (%), *DA* is the degree of acetylation of the chitosan derivative (%, *DA* = 100 − *DDA_SM_*), *M_mod-mono_* is the molecular weight of the modified repeating unit (g/mol), *m_Chit_* is the mass of the chitosan sample used in the titration (g), and Δ*M* is the difference between the molar mass of the modified repeating unit and the D-glucosamine repeating unit (g/mol).

**Figure 1:**
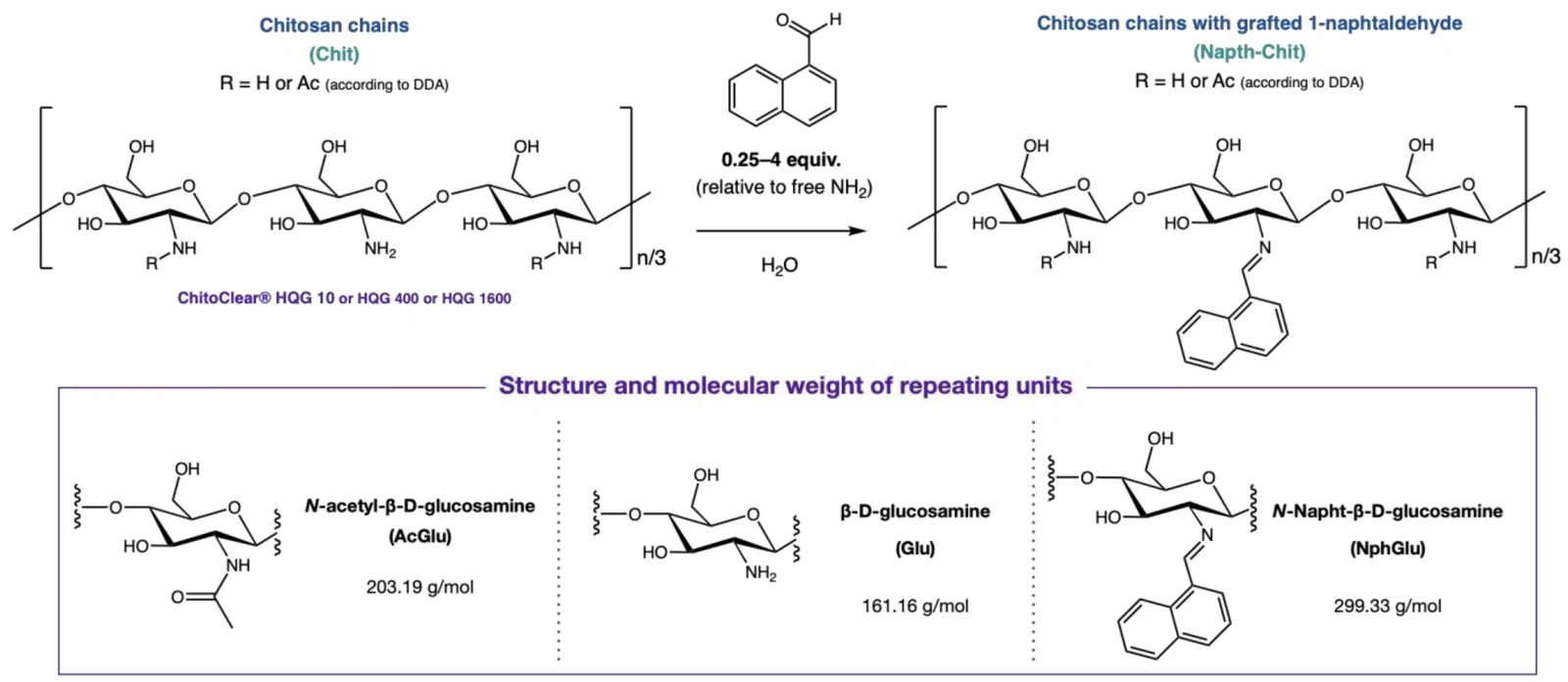
Preparation and structure of Napht-Chit derivatives.

In our previous study, we tested this equation by determining the DDA of a series of chitosan derivatives we prepared by grafting 1-naphthaldehyde to commercially available high-grade chitosans (ChitoClear^®^ HQG, Primex ehf, Siglufjörður, Iceland) [26]. The preparation and the structure of these chitosans, which we named Napth-Chit*,* are summarized in Figure 1.

Values for Napth-Chit derivatives can be substituted into Equation (2) to form Equation (3):

(3) $$DDA\;\left(\%\right)\;=100\frac{\left(∆V\cdot \left[\mathrm{NaOH}\right]\right)\left(\left(203.19\;g/\mathrm{mol}\cdot DA\right)+\left(299.33\;g/\mathrm{mol}\cdot DDA_{SM}\right)\right)}{m_{chit}+\left(138.17\;g/\mathrm{mol}\cdot ∆V\cdot \left[\mathrm{NaOH}\right]\right)}$$

for which Δ*V* is the difference between the two inflection points (L), [NaOH] is the concentration of the sodium hydroxide solution used for titration (mol/L), *DDA_SM_* is the degree of deacetylation (%) of the chitosan starting material, *DA* is the degree of acetylation of the chitosan derivative (%, *DA* = 100% − *DDA_SM_*), 203.19 g/mol is the molecular weight of the *N*-acetylglucosamine repeating unit, 299.33 g/mol is the molecular weight of the modified repeating unit (*N*-Napht-β-D-glucosamine), *m_Chit_* is the mass of the chitosan sample used in the titration (g), and 138.17 g/mol is the difference between the molar mass of the modified repeating unit and the D-glucosamine repeating unit.

In our previous work, we prepared fifteen different Napht-chit derivatives and submitted them to potentiometric titration [26]. The experimental data were treated using both Equations (1) and (3). As expected, for each modified chitosan, the obtained DDA was always higher when Equation (3) was used for data treatment, with a difference ranging from 2 to 5.4%. Since our equation is more in line with the real molecular composition of the modified chitosans, we hypothesize that it improved the accuracy of the potentiometric titration method for determining DDA [26].

However, to confirm this hypothesis, we needed to challenge our results by comparing them with a method of DDA determination known for its superior accuracy: ^1^H NMR.

## 3. Results and Discussion

To perform the comparison between potentiometric titration and ^1^H NMR, we selected the 10 previously synthesized Napht-Chit derivatives presented in Table 1. That selection gives a good overview of the variety (in terms of the length of the polymeric chain and the level of modification) of modified chitosans we explored in our previous study. To test the reproducibility of our protocol, we included a set of duplicates, i.e., two chitosans synthesized separately, but with the same reaction conditions (Table 1, entries 5 and 6).

**Figure 2:**
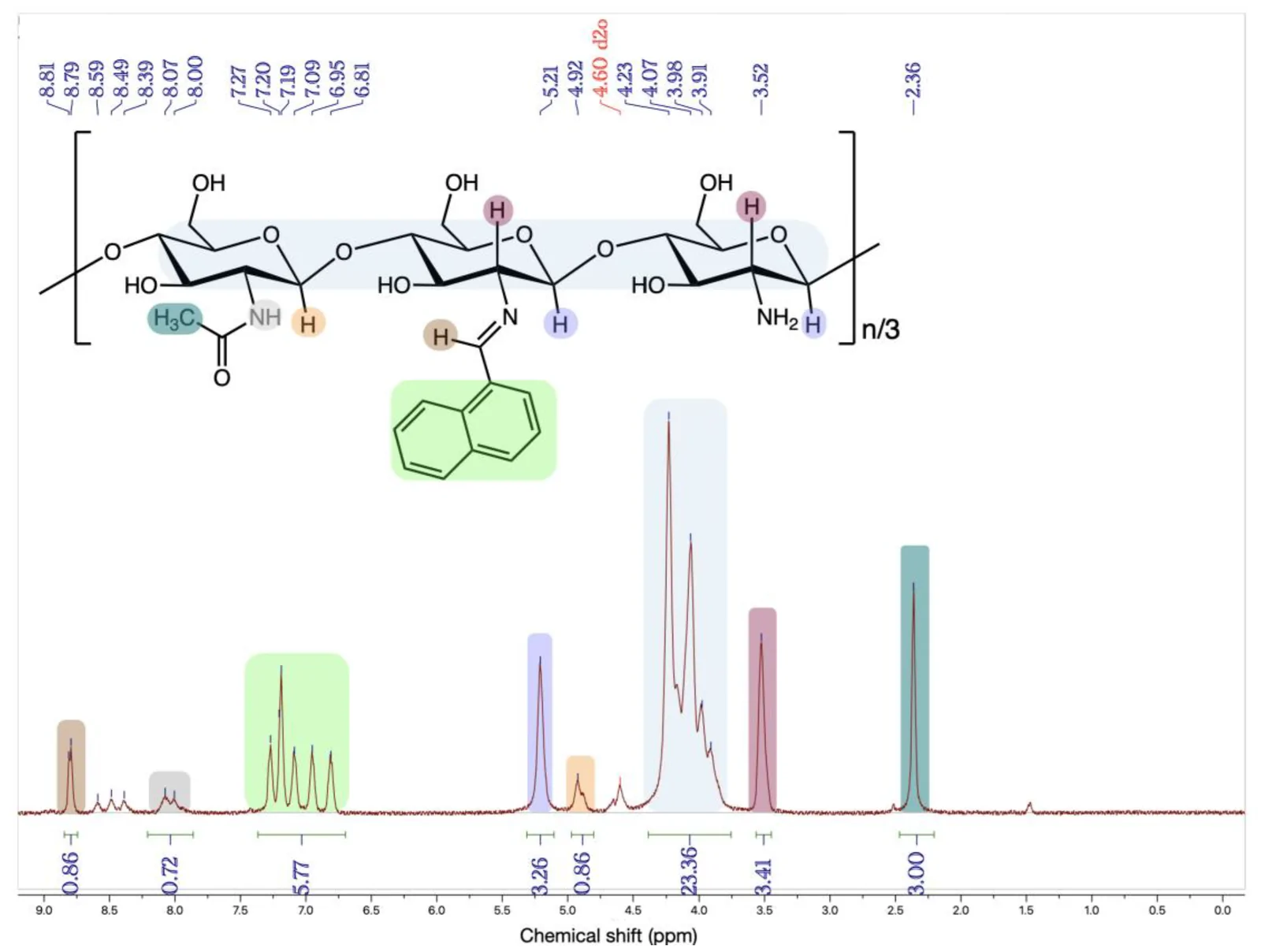
Illustration of a typical ^1^H NMR spectra for the Napht-Chit derivatives: NC-2d. Assignation: δ = 8.80 [H_im_]; 8.17–7.93 [H_NH-AcGlu_]; 7.33–6.75 [H_Ar_]; 5.21 [H_1-mix_ = H_1-glu_ + H_1-NphGlu_]; 4.92 [H_1-AcGlu_]; 4.36–3.80 [H_2-6-AcGlu_ + H_3-6-glu_ + H_3-6-NphGlu_]; 3.52 [H_2-glu_ + H_2-NphGlu_]; 2.36 [H_Ac_].

^1^H NMR spectrum for all products presented in Table 1 were recorded at 70 °C in a mixture of D_2_O/HCl, using an Agilent DD2 500 MHz instrument (see Supplementary Materials). As an example, Figure 2 shows the spectra recorded for NC-2d.

To determine the DDA of the chitosan derivatives by ^1^H NMR, we used three distinctive peaks, one belonging to each of the three repeating units (Table 2). For the *N*-acetyl-β-D-glucosamine repeating unit (AcGlu), we used the acetyl CH_3_ singlet whose chemical shift was previously reported at 2.36 ppm [17]. For D-glucosamine (Glu), we used the singlet associated with the anomeric proton at 5.21 ppm [17]. Finally, for the *N*-Napht-D-glucosamine repeating unit (NphGlu), we used the signal associated with the imine proton and centered at 8.80 ppm [27].

The integration values for those peaks on all the recorded spectra are detailed in Table 3. Note that the narrow singlet at 2.36 ppm (H_Ac_) was used as a reference. Its integration value was fixed at 3.00 on each spectrum.

The integration recorded for the peaks at 2.36 ppm and 8.80 ppm is directly linked to the ratio of the AcGlu and the NphGlu repeating units, respectively. However, the situation is different with the peak at 5.21 ppm, which was previously attributed to the β-D-glucosamine repeating unit. For that peak, the integration recorded on the spectrum (marked as H_1-mix_ in Table 3) cannot be used directly to calculate the ratio of the Glu repeating unit, because this peak also contains the signal originating from the anomeric proton of the NphGlu repeating unit (H_1-NphGlu_). Two reasons lead us to this conclusion. First, the closest example of this type of structure that we could find in the literature (a chitooligosaccharide bearing a salicylimine moiety) shows a chemical shift for its anomeric proton at 5.21 ppm [28]. Second, the only other peak in the anomeric region of our spectrum is positioned at 4.92 and corresponds to the anomeric proton of the AcGlu repeating unit [17]. Since its integration is always in about a 1:3 ratio with the H_Ac_ singlet of the same repeating unit (at 2.36 ppm), it is not possible that this peak also contains the signal for H_1-NphGlu_.

However, since it is expected that the integration of the imine proton (H_im_) and the anomeric proton (H_1-NphGlu_) are the same on the NphGlu repeating unit, the integration corresponding to the anomeric proton of the Glu repeating unit (*H_1-glu_*) can be obtained via Equation (4):

(4) $$H_{1-glu}=H_{1-mix}-H_{im}$$

for which *H*_1*-mix*_ is the integration recorded for the peak centered at 5.21 ppm on a ^1^H NMR spectrum, *H_im_* is the integration recorded for the peak centered at 8.80 ppm on the same ^1^H NMR spectrum, and *H*_1*-glu*_ is the calculated contribution of the anomeric proton of the Glu repeating unit on the *H*_1*-mix*_ signal.

With the integration values recorded in Table 3, we were able to determine the proportion of each repeating unit within the polymeric structure. Since there are three types of repeating units, we first established Equation (5):

(5) 100=%AcGlu+ %Glu+%NphGlu

for which %*AcGlu* is the percentage of the *N*-acetyl-β-D-glucosamine (AcGlu) repeating unit found in the polymeric chain, %*Glu* is the percentage of β-D-glucosamine (Glu) repeating unit found in the polymeric chain, and %*NphGlu* is the percentage of *N*-Napht-β-D-glucosamine repeating unit found in the polymeric chain.

The term %*AcGlu* can first be defined, because the *H_Ac_* signal is used as the reference for integration on the ^1^H NMR spectrum. Since the integration of this signal has been fixed to 3.00 and since it corresponds to three protons on the structure of the AcGlu repeating unit, we can express %AcGlu as in Equation (6):

(6) $$\%AcGlu=100\left(x\cdot \frac{H_{Ac}}{3}\right)=100x$$

for which %*AcGlu* is the percentage of the *N*-acetyl-β-D-glucosamine (AcGlu) repeating unit found in the polymeric chain, *x* is the molar ratio of the *N*-acetyl-β-D-glucosamine (AcGlu) repeating unit found in the polymeric chain, and *H_Ac_* is the integration recorded on the ^1^H spectrum for the peak located at 2.36 ppm.

From there, since *H_Ac_* is used as the integration reference on the NMR spectrum, %*Glu* and %*NphGlu* can also be defined as a factor of *x*. This yields Equations (7) and (8):

(7) $$\%Glu=100\left(x\cdot H_{1-glu}\right)$$

(8) $$\%NphGlu=100\left(x\cdot H_{Im}\right)$$

for which %*Glu* is the percentage of the β-D-glucosamine (Glu) repeating unit found in the polymeric chain, *x* is the molar ratio of the *N*-acetyl-β-D-glucosamine repeating unit (AcGlu) found in the polymeric chain, %*NphGlu* is the percentage of *N*-Napht-β-D-glucosamine repeating unit (NphGlu) found in the polymeric chain, *H*_1*-glu*_ is the contribution of the anomeric proton of the Glu repeating unit on the signal recorded on the ^1^H NMR at 5.21 ppm, and *H_im_* is the integration recorded for the peak centered at 8.80 ppm on the same ^1^H NMR spectrum.

Substituting the terms %*AcGlu*, %*Glu* and %*NphGlu* in Equation (5) by using Equations (6)–(8), leads us to Equation (9), which, simplified, yields Equation (10):

(9) $$100=100x+\;100\left(x\cdot H_{1-glu}\right)+100\left(x\cdot H_{Im}\right)$$

(10) $$1=x\;\left(1+H_{1-glu}+H_{Im}\right)$$

for which *x* is the molar ratio of the AcGlu repeating unit found in the polymeric chain, *H*_1*-glu*_ is the contribution of the anomeric proton of the Glu repeating unit on the signal recorded on the ^1^H NMR at 5.21 ppm, and *H_im_* is the integration recorded for the peak centered at 8.8 ppm on the same ^1^H NMR spectrum.

From there, the variable *x* can be isolated and calculated using Equation (11):

(11) $$x=\;\frac{1}{1+\;H_{1-glu}+\;H_{Im}}$$

Once variable *x* is calculated with Equation (11), the respective proportions of the three repeating units for a given sample can be obtained using Equations (6)–(8). Table 4 presents the distribution of repeating units within each studied chitosan derivative.

The results presented in Table 4 show several interesting points. First, Table 4 can attest to the reproducibility of both our method of chitosan modification and ^1^H NMR analysis; we know this because the results obtained for NC-2cR1 and NC-2cR2, two chitosans synthesized in parallel under the same exact conditions, gave comparable results (entry 5 vs. entry 6).

Second, we observed that the %AcGlu is consistent for all the NC-1 derivatives (Table 4, entries 1–3) with an average and standard deviation of 22% ± 1. The same can be said for all the NC-2 derivatives (entries 4–8: 22.6% ± 0.5) and the NC-3 derivatives (entries 9–10: 29.5% ± 0.5). This was expected since a common starting material was used for each series (see Table 1 for details) and the proportion of this repeating unit is not supposed to be affected by the chemical modification those chitosans were subjected to.

Third, our ^1^H NMR analysis allowed us to better understand the amination reaction used to modify those chitosan derivatives. As expected, we observed that the use of a larger excess of 1-naphthaldehyde during the reaction leads to more modification within the polymer chain (higher %NphGlu). For example, for the NC-1 series (Table 4, entries 1–3), we observed that the values of %NphGlu evolved from 6% to 22% as the Napht:NH_2_ (1-naphthaldehyde to free amines on chitosan) stoichiometric ratio used for the chemical modification increased from 1:2 to 4:1. Interestingly, we observed that the three chitosan starting materials behave quite differently in the context of imination with 1-naphthaldehyde. Table 4 shows that a Napht:NH_2_ ratio of 1:2 was sufficient to obtain a value of %NphGlu of 18% for the NC-3 series (entry 9), while a ratio of 1:1 was needed to achieve the same %NphGlu for the NC-2 series (entry 5). For the NC-1 series, a ratio of 4:1 was necessary to reach a value of %NphGlu of the same order of magnitude (Table 4, entry 3). Overall, those results suggested that the longer the polymeric chain, the easier it is to graft naphthalene groups on it. Our preliminary hypothesis to explain this trend is that the shorter chains tend to agglomerate more easily, thereby hindering access to the amino functionality.

Finally, Table 4 shows the DDA (listed as %Glu) determined by ^1^H NMR, allowing us to reach the main goal of this study: the comparison between the DDA obtained by potentiometric titration and by ^1^H NMR (*DDA_NMR_*). To facilitate this comparison, Table 5 regroups the DDA values we obtained by potentiometric titration (*DDA_PT_*) in our previous study [26] with the DDA values obtained by ^1^H NMR (*DDA_NMR_*) in the current study.

Table 5 shows that the DDA obtained by potentiometric titration will vary according to the equation used to treat the raw titration data. As previously mentioned, for each chitosan derivative, the DDA obtained with the classic Broussignac equation (*DDA*_*PT*_*Eq*___1_) was lower than the one obtained with our own equation specifically suited for modified chitosan (*DDA*_*PT*_*Eq*___3_). Overall, Table 5 shows that those later values tend to be closer to the one we just determined by ^1^H NMR (*DDA_NMR_*).

To emphasize this trend, Table 6 presents the data in terms of ΔDDA, i.e., the deviation from the ^1^H NMR value of the DDA value obtained by potentiometric titration. Equations (12) and (13) define two variants of ΔDDA, depending on the equation used to determine the DDA by potentiometric titration:

(12) $$\Delta DDA_{Eq\_1}=DDA_{NMR}-DDA_{PT\_Eq\_1}$$

(13) $$\Delta DDA_{Eq\_3}=DDA_{NMR}-DDA_{PT\_Eq\_3}$$

for which *DDA*_*PT*_*Eq*___1_ is the DDA obtained by potentiometric titration with Equation (1), *DDA*_*PT*_*Eq*___3_ is the *DDA* obtained by potentiometric titration with Equation (3), and *DDA_NMR_* is the DDA obtained by ^1^H NMR.

Also, the term “difference in deviation from ^1^H NMR value (Δ*Dev*)” is introduced in Table 6. This term is useful to demonstrate, in terms of DDA units (in % by definition), that Equations (1) and (3) can yield different DDA values that have a different deviation from the value obtained by ^1^H NMR. It is defined by Equation (14):

(14) $$\Delta Dev\;\left(\%\right)=\left|\Delta DDA_{Eq1}\right|-\;\left|\Delta DDA_{Eq3}\right|$$

Table 6 shows that for eight chitosans, Equation (3) provided a DDA value that was closer to that obtained by ^1^H NMR (entries 1, 2, 4–7, and 9–10). The positive value of Δ*Dev* recorded for those chitosans explicitly illustrates that Equation (3) give a DDA value that is more in agreement with the value determined by ^1^H NMR. For two cases, there was no difference between Equations (1) and (3), resulting in an Δ*Dev* value of 0 for those two derivatives (Table 6, entries 3 and 8). Interestingly, we observed for those two cases that Equation (3) was slightly overestimating the DDA, while Equation (1) was underestimating it at the same level.

We found that those results were quite surprising, as the potentiometric titration protocol we used is usually considered to have an underestimation bias [15]. In fact, Table 6 shows that using Equation (3) to determine the DDA by potentiometric titration overestimates the DDA for 6 cases out of 10. Although this might be seen as a new drawback introduced by the use of Equation (3), we observed that having positive and negative Δ*DDA*_*Eq*___3_ values yields an overall more tightly clustered distribution of the DDA obtained by potentiometric titration around the targeted value (the DDA obtained by ^1^H NMR). Indeed, in 7 cases out of 10, Δ*DDA*_*Eq*___3_ ranged between 0 and −2 DDA units (Table 6, entries 3–7, 9 and 10). In 8 cases out of 10, it ranged between 0 and −3 DDA units (Table 6, entries 3–10).

**Figure 3:**
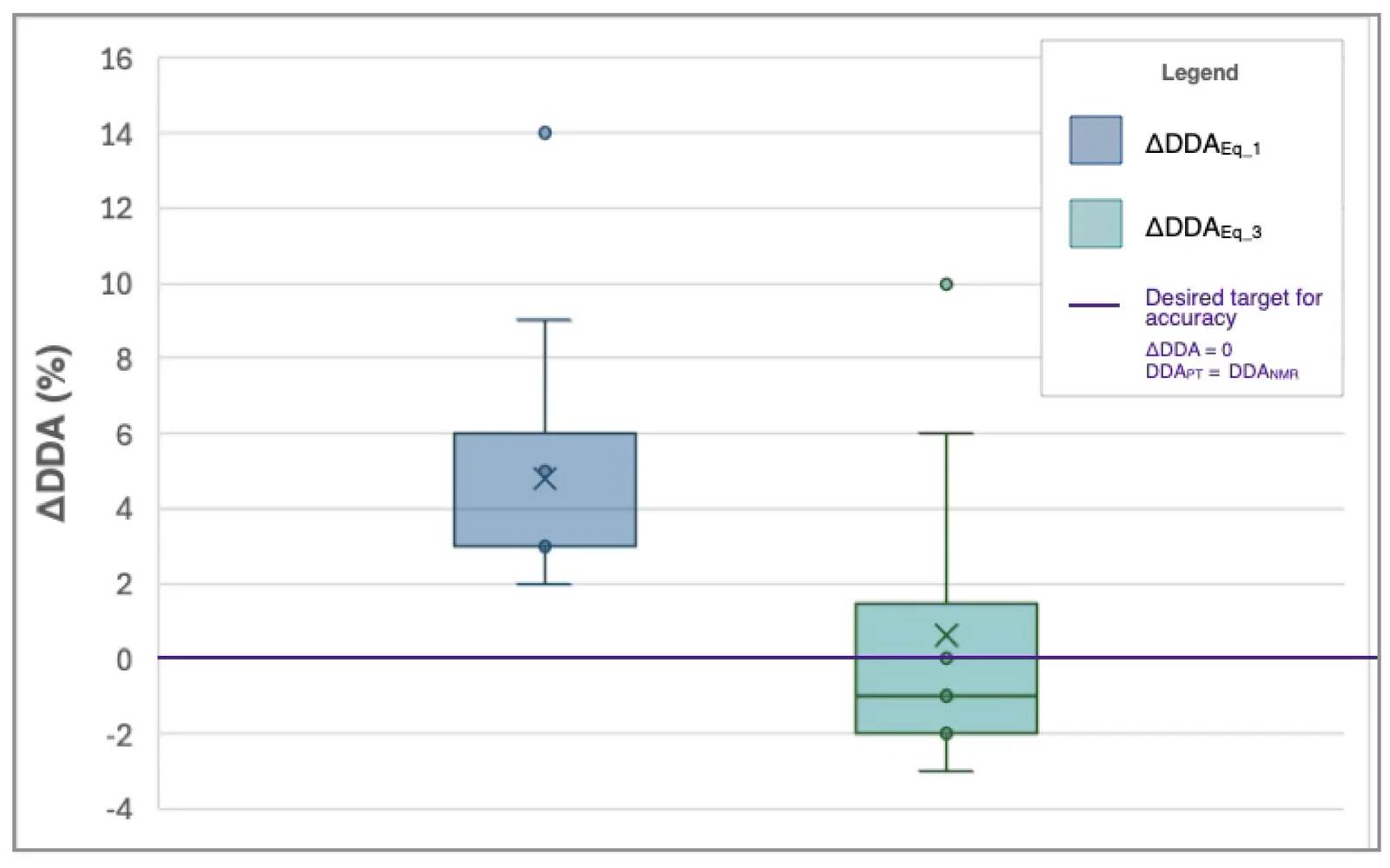
Box and whisker plot showing the distribution the values ofΔ*DDA*_*Eq*___1_ (blue) and Δ*DDA*_*Eq*___3_ (teal) obtained for the 10 studied chitosan derivatives. The median of each distribution is marked by an X.

Figure 3, which regroups the values of Δ*DDA*_*Eq*___1_ and Δ*DDA*_*Eq*___3_ obtained for all the chitosan derivatives in a box-and-whisker plot, illustrates that observation. Indeed, this plot clearly shows that the values of Δ*DDA*_*Eq*___3_ obtained for all the chitosans involved in this study form an ensemble that is less dispersed and much closer to the desired target than the one formed with the values of Δ*DDA*_*Eq*___1_ obtained for the same chitosan derivative. The desired target here (marked by a purple line in Figure 3) corresponds to a Δ*DDA* value of 0, which means a perfect match between the DDA determined by potentiometric titration and the one determined by NMR.

## 4. Materials and Methods

### 4.1. Preparation of Napth-Chit Derivatives

We detailed our preparation methods and FT-IR characterizations for the Napht-Chit derivatives used in this study, in our previous study [26]. In short, 1 g of lyophilized chitosan (ChitoClear^®^ HQG 10 or ChitoClear^®^ HQG 400 or ChitoClear^®^ HQG 1600; Primex ehf, Siglufjörður, Iceland) was added to 5 mL of distilled water in a conical centrifuge tube. Then, 4 mL of ethanol was added to each tube to solubilize the 1-naphtaldehyde. Next, a certain volume (calculated to reach the desired Napht:NH_2_ ratio and rounded to the closest 0.1 mL) of 1-naphtaldehyde (Sigma-Aldrich, Mississauga, ON, Canada) was added. Then water was added to reach a total volume of 20 mL. The reaction was stirred for 48 h at room temperature and then centrifuged at 3400 g for 6 min. The settled modified chitosan was rinsed 9 times with a volume of 40 mL of [sodium chloride 30 g/L (3×), distilled water (2×), acetone (1×), ethanol (1×), and distilled water (×2)] (with a 5 min agitation followed by centrifugation at 3400 g for 6 min). After the ninth rinse, the supernatant water was removed, and the remaining solid was lyophilized.

### 4.2. Potentiometric Titration of Chitosan Derivatives

We also published the detailed protocol for the titration of the Napht-Chit derivatives in our previous study [26]. In short, a solution of 50 mg of previously lyophilized chitosan derivative in 20 mL of diluted HCl (0.03 mol/L) was prepared and stirred for 30 min. The resulting solution was then titrated with NaOH solution (0.1 mol/L). During titration, the volumes of NaOH added and the pH values of the solution were monitored with an automatic titrator (848 Titrino plus, Metrohm, Herisau, Switzerland). Prior to each titration, we calibrated the pH electrode with buffer solutions of pH 4.00 and 7.00.

### 4.3. NMR Analysis of a Chitosan Derivative

We added 1.5 mL of D_2_O (Sigma-Aldrich, ON, Canada) and 10 μL of conc. HCl to a sample of chitosan (25 mg) in a closed 8 mL vial. The resulting suspension was stirred at room temperature for 24 h and was then roughly filtered through cotton directly into a 5 mm NMR tube. A ^1^H NMR spectrum was acquired at 70 °C on an Agilent DD2 500 MHz NMR using Agilent OneNMR probe (Agilent Technologies, Santa Clara, CA, USA). The experiment consisted of a single pulse sequence with presaturated solvent (D_2_O/HOD). The presaturation step was achieved by a 2 s pulse, with a power of 21 Hz, at a 4.67 ppm frequency. This was followed by an 8.5 μs 90° pulse (pw90) with a power of 58,824 Hz. A relaxation delay of 2 s was given to the sample between each acquisition. The spectral width was 8013 Hz, the number of data points was 32,768, the Fourier number was 65,536, and no line broadening was used. Each experiment was composed of 8 scans, which resulted in a total acquisition time of approximately 5 min per sample, including equilibration and shimming. MestReNova (version 14.3.1, Mestrelab Research S.L, Santiago de Compostela, Spain) was used to process the FID signal and perform baseline correction (Berstein Polynomial fit, order 6). The singlet of the *N*-acetyl-D-glucosamine repeating unit was used as the chemical shift (Δ = 2.36) and integration (*I* = 3) reference.

## 5. Conclusions

In summary, we continued our study of a series of our previously reported naphthalene-chitosan derivatives by determining their repeating unit composition by ^1^H NMR. In addition to providing us with useful insights on the imination reaction used for their modification, the current study allowed us to challenge the DDA values we previously obtained for those chitosans by potentiometric titration.

In our previous study, we calculated the DDA of those chitosan derivatives from data obtained by titration using two equations: the classical Broussignac equation and a new equation we developed that is specifically suited for modified chitosans that bear three types of repeating units. The two equations gave different DDA values for each derivative we studied. In the current study, we wanted to confirm that our equation gave DDA values more comparable than what can be obtained by ^1^H NMR, thus proving its utility to provide results more in agreement with the spectroscopic method generally regarded as the more accurate. We observed that for 8 out of 10 samples, our equation provided a DDA value that was closer (between 1 and 5 DDA units) to the one obtained by ^1^H NMR; and for 2 samples, the two equations provided an identical deviation from the NMR value.

In conclusion, this study demonstrates that, by relying on an equation that better characterizes the molecular structure of the polymer chains, potentiometric titration can indeed be almost as accurate as ^1^H NMR to determine the degree of deacetylation of modified chitosan. From a wider perspective, the results reported in this paper demonstrate that revisiting and refining the data processing stage associated with a traditional analytical technique can help to attenuate its limitations. Since the current demonstration only involves a specific series of 10 naphthalene–chitosan derivatives, we are currently working on the preparation and characterization of a new series of chitosan derivatives to confirm that our conclusion could be generalized to a wider selection of modified chitosans.

## Figures and Tables

**Table 1:** Complete description of the chitosan derivatives involved in this study: starting material used, naphthaldehyde–free amine ratio (Napht:NH_2_) used for modification, name of the product.

Entry	Starting Material Name [MW of Polymer Chains (kDa)] ^I^	Napht:NH_2_^II^	Product Name ^IV^	Abbreviated Name ^V^
1	ChitoClear^®^ HQG 10 [110–150]	1:2	Napht-Chit-1b	NC-1b
2		2:1	Napht-Chit-1d	NC-1d
3		4:1	Napht-Chit-1e	NC-1e
4	ChitoClear^®^ HQG 400 [250–300]	1:4	Napht-Chit-2a	NC-2a
5		1:1 ^III^	Napht-Chit-2c (R1) ^III^	NC-2cR1
6		1:1 ^III^	Napht-Chit-2c (R2) ^III^	NC-2cR2
7		2:1	Napht-Chit-2d	NC-2d
8		4:1	Napht-Chit-2e	NC-2e
9	ChitoClear^®^ HQG 1600 [340–360]	1:2	Napht-Chit-3b	NC-3b
10		1:1	Napht-Chit-3c	NC-3c

I. See reference [26] for other characterizations: IR, viscosity, and DDA. II. Ratio of 1-naphthaldehyde to free amines (Napht:NH_2_ ratio) used for chemical modification. III. Two replicates (R1 and R2) synthesized separately. IV. Names used in our previous work [26]. V. Names used in the current manuscript.

**Table 2:** Key signals used to determine the composition of each repeating unit on the chitosan derivatives from the ^1^H NMR spectra.

Monomer	Proton Use for NMR Analysis (Marked in Red)		δ (ppm)
*N*-acetyl-β-D-glucosamine (AcGlu)	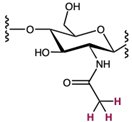	**H_Ac_**	2.36
β-D-glucosamine (Glu)	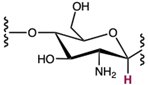	**H_1-glu_**	5.21
*N*-Napht-β-D-glucosamine (NphGlu)	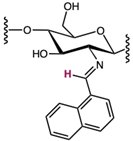	**H_im_**	8.80

**Table 3:** Integration for peaks of interest recorded on the ^1^H NMR spectra of all studied modified chitosans.

Chitosan Derivative	Integration Recorded on the ^1^H NMR Spectra ^III^			
	H_Ac_^I^ δ = 2.36 ppm	H_1-mix_ δ = 5.21 ppm	H_1-glu_^II^ δ = 5.21 ppm	H_im_ δ = 8.80 pm
NC-1b	3.00	3.29	3.03	0.26
NC-1d	3.00	3.51	3.19	0.32
NC-1e	3.00	3.70	2.67	1.03
NC-2a	3.00	3.35	3.10	0.25
NC-2cR1	3.00	3.45	2,66	0.79
NC-2cR2	3.00	3.50	2.59	0.91
NC-2d	3.00	3.26	2.40	0.86
NC-2e	3.00	3.44	2.28	1.16
NC-3b	3.00	2.35	1.75	0.60
NC-3c	3.00	2.50	1.71	0.79

I. Used as integration reference. II. Calculated using Equation (4). III. Complete spectra can be found in the Supplementary Materials.

**Table 4:** Repeating unit distribution within the polymeric chain of a series of modified chitosans, as determined by ^1^H NMR.

Entry	Chitosan Derivative	Repeating Unit Distribution in the Polymeric Chain (%)		
		%AcGlu	%Glu ^I^	%NphGlu
1	NC-1b	23	71	6
2	NC-1d	22	71	7
3	NC-1e	21	57	22
4	NC-2a	23	71	6
5	NC-2cR1	22	60	18
6	NC-2cR2	22	58	20
7	NC-2d	23	56	20
8	NC-2e	23	51	26
9	NC-3b	30	52	18
10	NC-3c	29	49	22

I. Could also be expressed as *DDA_NMR_* (%).

**Table 5:** DDA values of a series of modified chitosan calculated by potentiometric titration [*DDA_PT_* (%)] using two different equations, and by ^1^H NMR [*DDA_NMR_* (%)].

Entry	Chitosan Derivative	*DDA_PT_* (%)		*DDA_NMR_* (%)
		Classic Equation *DDA*_*PT*_*Eq*___1_ (%) ^I^	Our Equation *DDA*_*PT*_*Eq*___3_ (%) ^I^	
1	NC-1b	62	65	71
2	NC-1d	57	61	71
3	NC-1e	55	59	57
4	NC-2a	68	71	71
5	NC-2cR1	57	61	60
6	NC-2cR2	55	60	58
7	NC-2d	51	56	56
8	NC-2e	48	54	51
9	NC-3b	49	53	52
10	NC-3c	46	50	49

I. Rounded to the nearest unit (See the Supplementary Materials for detailed results).

**Table 6:** Difference between the *DDA* obtained by ^1^H NMR and the DDA obtained with potentiometric titration for either Equation (1) (Δ*DDA*_*Eq*___1_) or Equation (3) (Δ*DDA*_*Eq*___3_), and the difference in the deviation from the ^1^H NMR value between the two equations (Δ*Dev*).

Entry	Chitosan Derivative	Δ*DDA*_*Eq*___1_ (%)	Δ*DDA*_*Eq*___3_ (%)	Δ*Dev* (%)
1	NC-1b	9	6	+3
2	NC-1d	14	10	+4
3	NC-1e	2	−2	0
4	NC-2a	3	0	+3
5	NC-2cR1	3	−1	+2
6	NC-2cR2	3	−2	+1
7	NC-2d	5	0	+5
8	NC-2e	3	−3	0
9	NC-3b	3	−1	+2
10	NC-3c	3	−1	+2

## Data Availability

All the data discussed in this study are available either in the current manuscript, its Supplementary Materials, or a previously reported study (Ref. [26]).

## Supplementary Materials

The following supporting information can be downloaded at: https://www.mdpi.com/article/10.3390/molecules31213892/s1. S1. Determination of DDA by potentiometric titration: detailed values for the chitosan resins involved in this study. S2. Characterization of Napht-Chit chitosan derivatives (^1^H NMR and IR).
