# Supplementary material for: Can Potentiometric Titration Be Almost as Accurate as 1H NMR to Determine the Degree of Deacetylation of Modified Chitosan?
Source: Molecules. 2026 Oct 28;31(21):3892. doi: 10.3390/molecules31213892 (PMC13764783; doi:10.3390/molecules31213892)
Supplement: Supplementary file 1 [file molecules-31-03892-s001.zip › molecules-4609003-supplementary.pdf]

Supplementary materials

Can potentiometric titration be almost as accurate as ^{1}H NMR to determine the degree of deacetylation of modified chitosan?

Sébastien Cardinal[1,*], Tommy Fraser[1,2], and Sarah Kefil[1]

[1] Département de Biologie, Chimie et Géographie, Université du Québec à Rimouski, Rimouski, QC G5L 3A1, Canada
[2] Département de Chimie, Centre d'Études Nordiques and PROTEO, Université Laval, Québec, QC, G1V 0A6, Canada

*Correspondence: sebastien_cardinal@uqar.ca;

S1. Determination of DDA by potentiometric titration: detailed values for the chitosan resins involved in this study

Table S1: Determination of DDA by potentiometric titration: detailed values for the chitosan resins involved in this study*

Chitosan resin	Classic equation $DDA_{PT\_Eq\_1}$ (%)	Our equation $DDA_{PT\_Eq\_3}$ (%)
NC-1b	61.68	64.71 ± 0.04
NC-1d	56.73	60.62 ± 0.04
NC-1e	54.83	59.00 ± 0.04
NC-2a	68.50	70.7 ± 0.2
NC-2cR1	56.99	61.3 ± 0.1
NC-2cR2	54.99	59.6 ± 0.1
NC-2d	51.00	56.1 ± 0.1
NC-2e	48.20	53.6 ± 0.1
NC-3b	48.86	52.73 ± 0.04
NC-3c	45.59	49.83 ± 0.03

*Data reproduced from: Amamou, O.; Kefil, S.; Denis, J.-P.; Boubaker, T.; Cardinal, S. Revisiting the Determination of the Degree of Deacetylation Using Potentiometric Titration: A New Equation for Modified Chitosan. *Molecules* **2024**, *29*, 2962. https://doi.org/10.3390/molecules29132962

S2. Characterization of Napht-Chit chitosan derivatives (^{1}H NMR and IR).

S2.1 General remarks

S2.1.1 ^{1}H NMR analysis (protocol)

We added 1.5 mL of D_2O (Sigma-Aldrich) and 10 µL of conc. HCl to a sample of chitosan (25 mg) in a closed 8 mL vial. The resulting suspension was stirred at room temperature for 24 hours and was then roughly filtered through cotton directly into a 5 mm NMR tube. A ^{1}H NMR spectrum was acquired at 70 °C on an Agilent DD2 500 MHz NMR using Agilent OneNMR probe. The experiment consisted of a single pulse sequence with presaturated solvent (D_2O/HOD). The presaturation step was achieved by a 2 second pulse, with a power of 21 Hz, at a 4.67 ppm frequency. This was followed by an 8.5 µs 90° pulse (pw90) with a power of 58824 Hz. A relaxation delay of 2 seconds was given to the sample between each acquisition. The spectral width was 8013 Hz, the number of data points was 32768, the Fourier number was 65536, and no line broadening was used. Each experiment was composed of 8 scans, which resulted in a total acquisition time of approximately 5 minutes per sample, including equilibration and shimming. MestReNova (version 14.3.1, Mestrelab Research S.L) was used to process the FID signal and perform baseline correction (Berstein Polynomial fit, order 6). The singlet of the *N*-acetyl-D-glucosamine repeating unit was used as the chemical shift (δ = 2.36) and integration (I = 3) reference.

S2.1.2 FT-IR

Napht-Chit resins were analyzed by ATR-FTIR using a Nicolet iS50 instrument (Thermo Scientific, USA). A total of 32 scans were realized for each sample at a resolution of 4 cm 1. OMNIC spectra software (Thermo Scientific, USA) was used to normalize the spectra and investigate the absorbance bands.

Four bands were used to confirm the formation of the imine functionalities between 1-naphtaldehyde and chitosan. Those four bands were absent from the IR spectrum of the starting materials. Furthermore, those bands become more defined and intense as the level of functionalization increases. The first two bands around 770 and 800 cm^{-1} are attributed to the aromatic out-of-plane C-H bending. The third band around 1500 cm^{-1} is associated with C=C ring stretch. The fourth band around 1675 cm^{-1} is attributed to the C=N bond of the imine functionality. It is a bit more challenging to observe because unmodified chitosan shows a strong peak around 1645 cm^{-1} associated with the residual N-acetyl groups (C=O stretching of amide I).

S2.2 Characterization of NC-1b

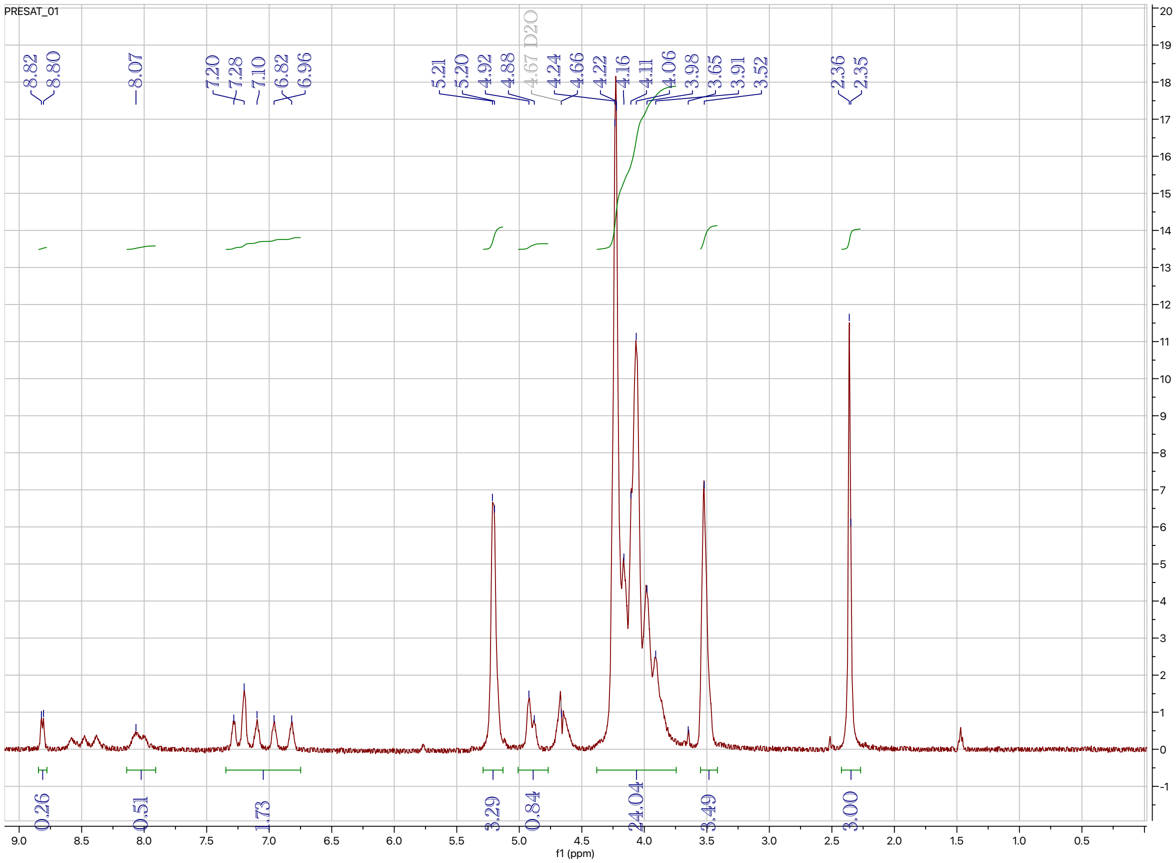

Figure S1. ^{1}H NMR spectra for chitosan derivative NC-1b.

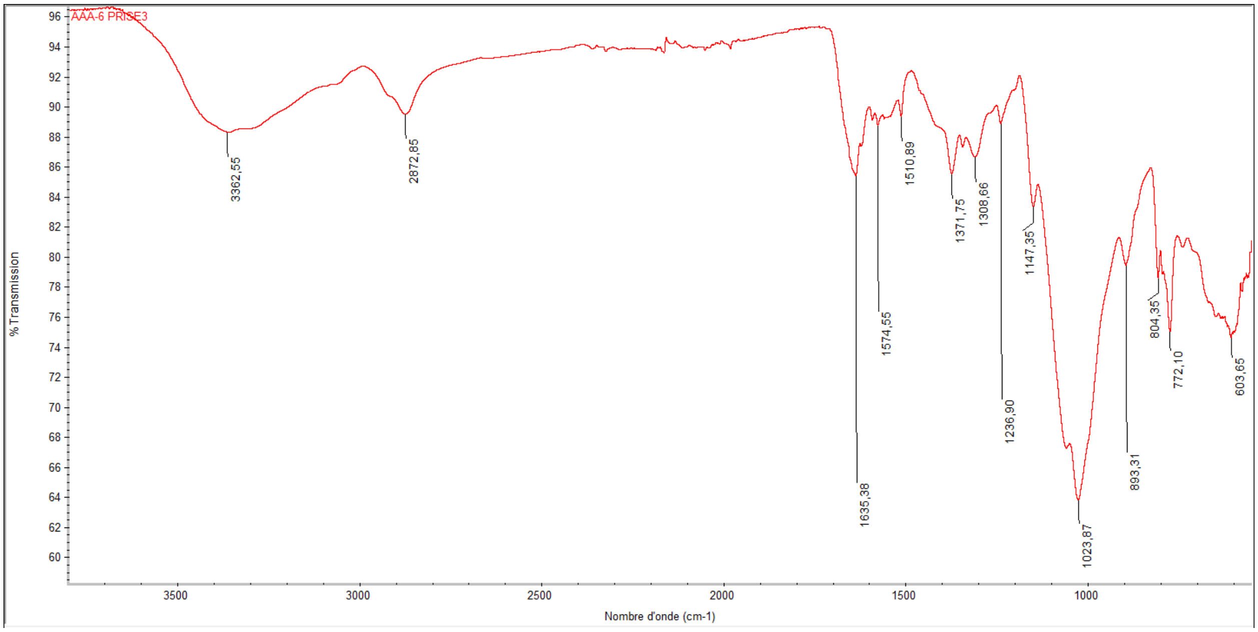

Figure S2. FTIR spectra for chitosan derivative NC-1b

S2.3 Characterization of NC-1d

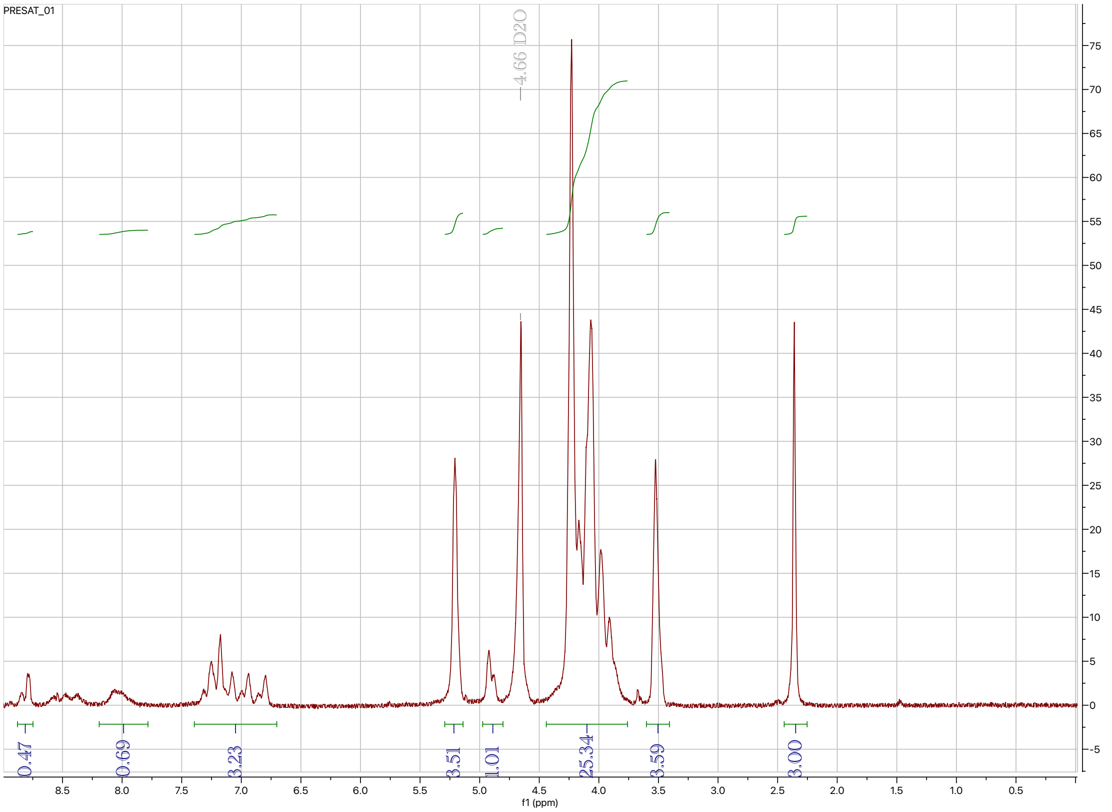

Figure S3. ^{1}H NMR spectra for chitosan derivative NC-1d.

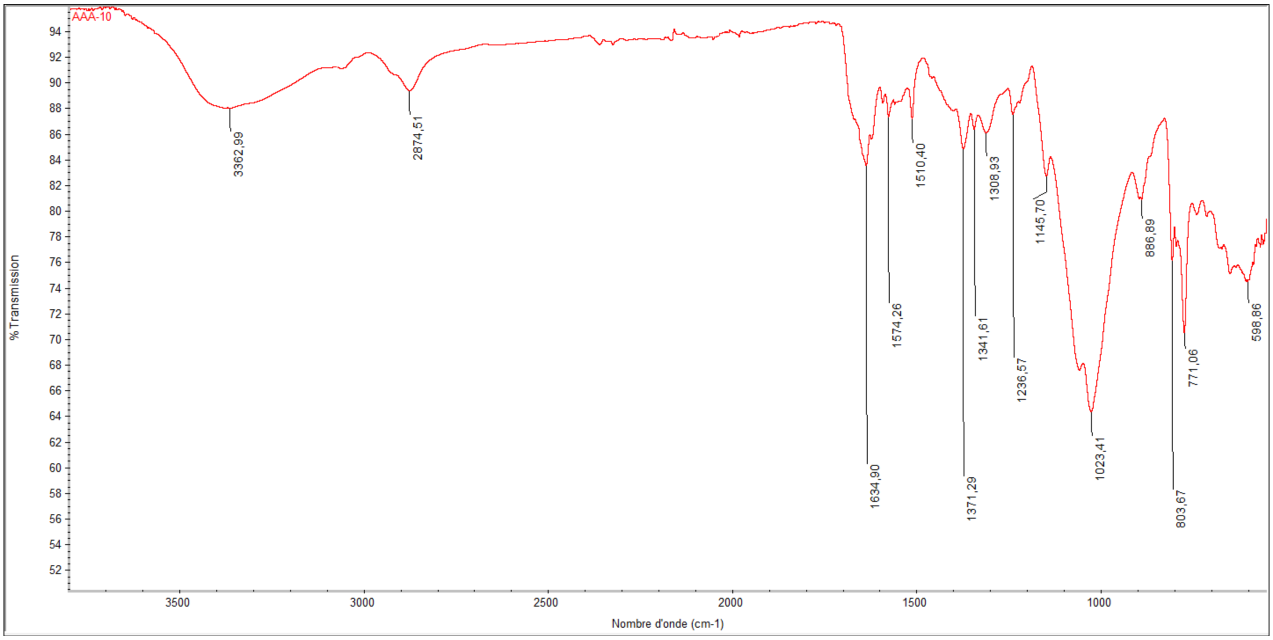

Figure S4. FTIR spectra for chitosan derivative NC-1d.

S2.4 Characterization of NC-1e

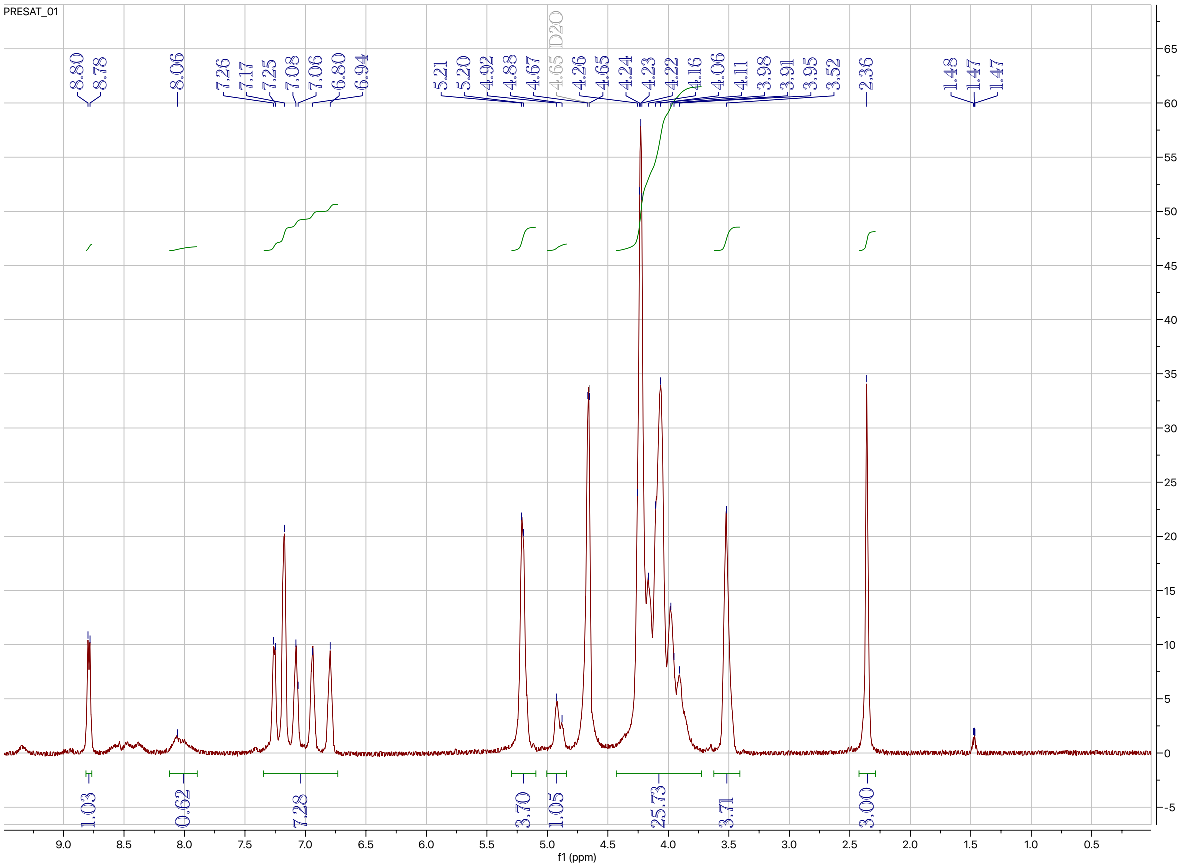

Figure S5. ^{1}H NMR spectra for chitosan derivative NC-1e.

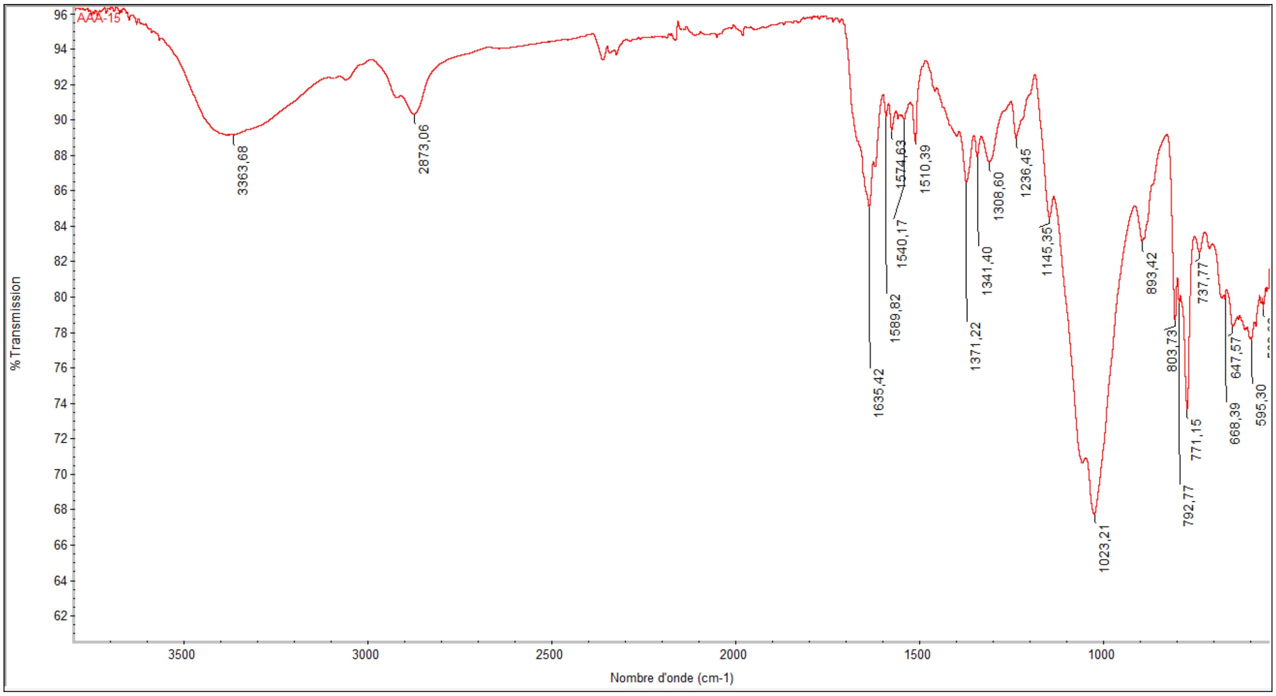

Figure S6. FTIR spectra for chitosan derivative NC-1e.

S2.5 Characterization of NC-2a

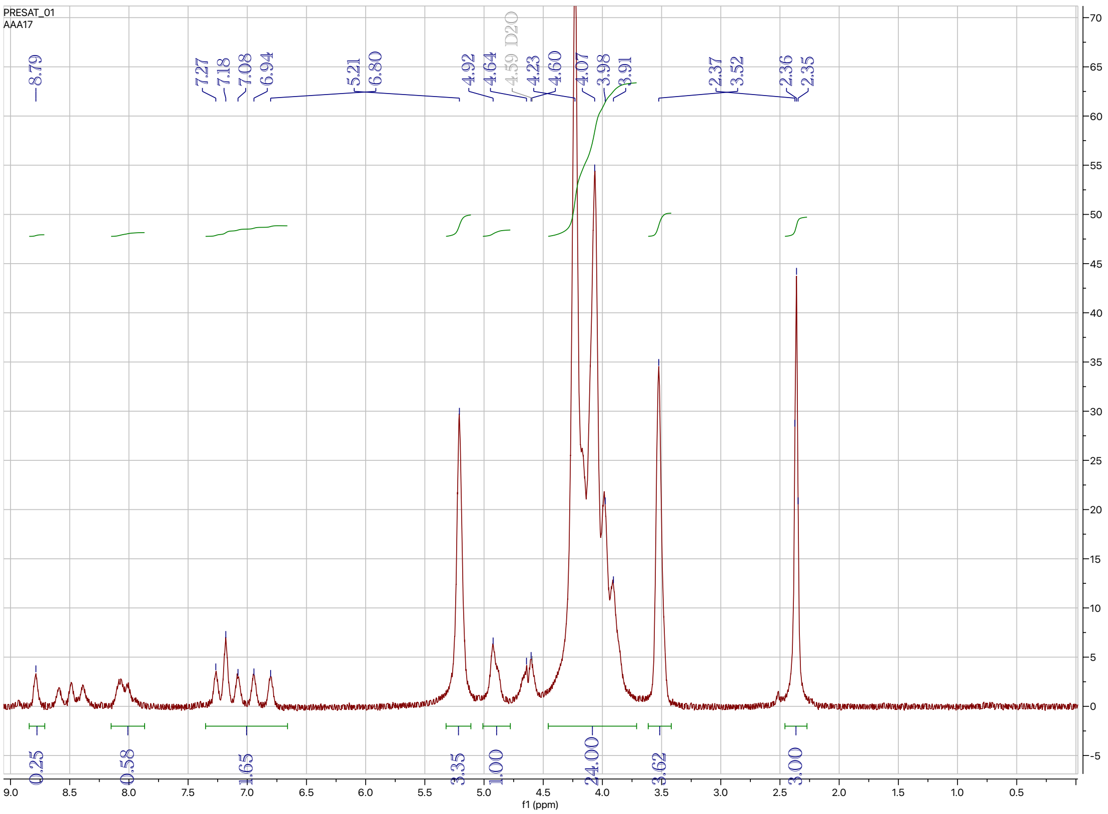

Figure S7. ^{1}H NMR spectra for chitosan derivative NC-2a.

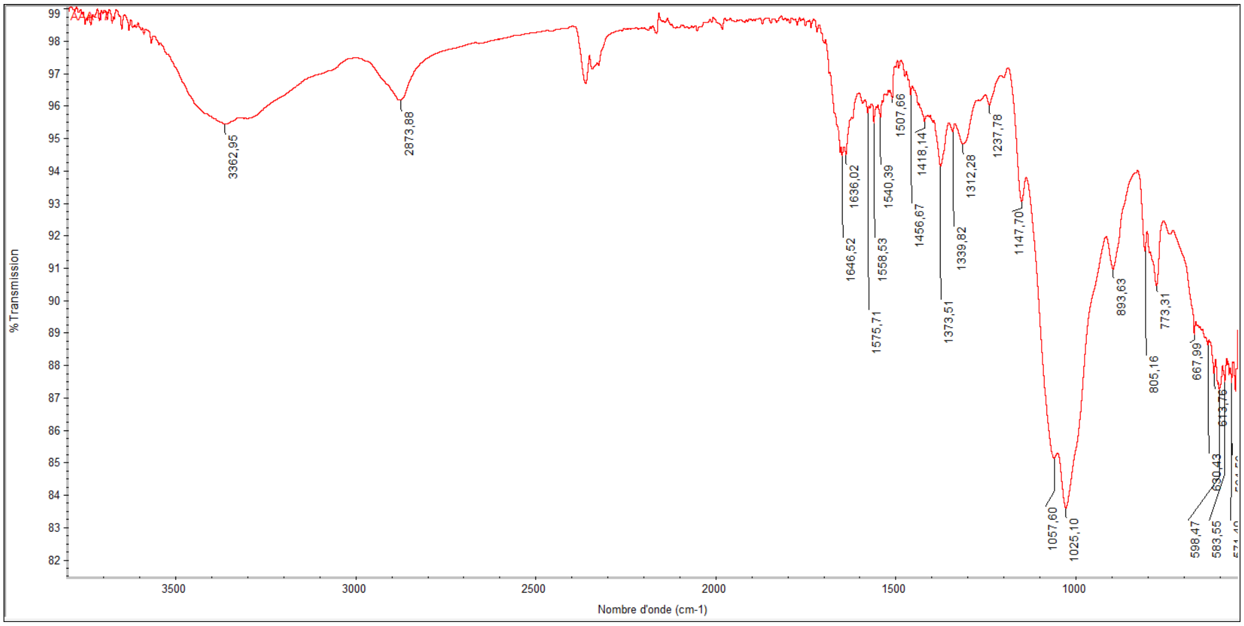

Figure S8. FTIR spectra for chitosan derivative NC-2a.

S2.6 Characterization of NC-2cR1

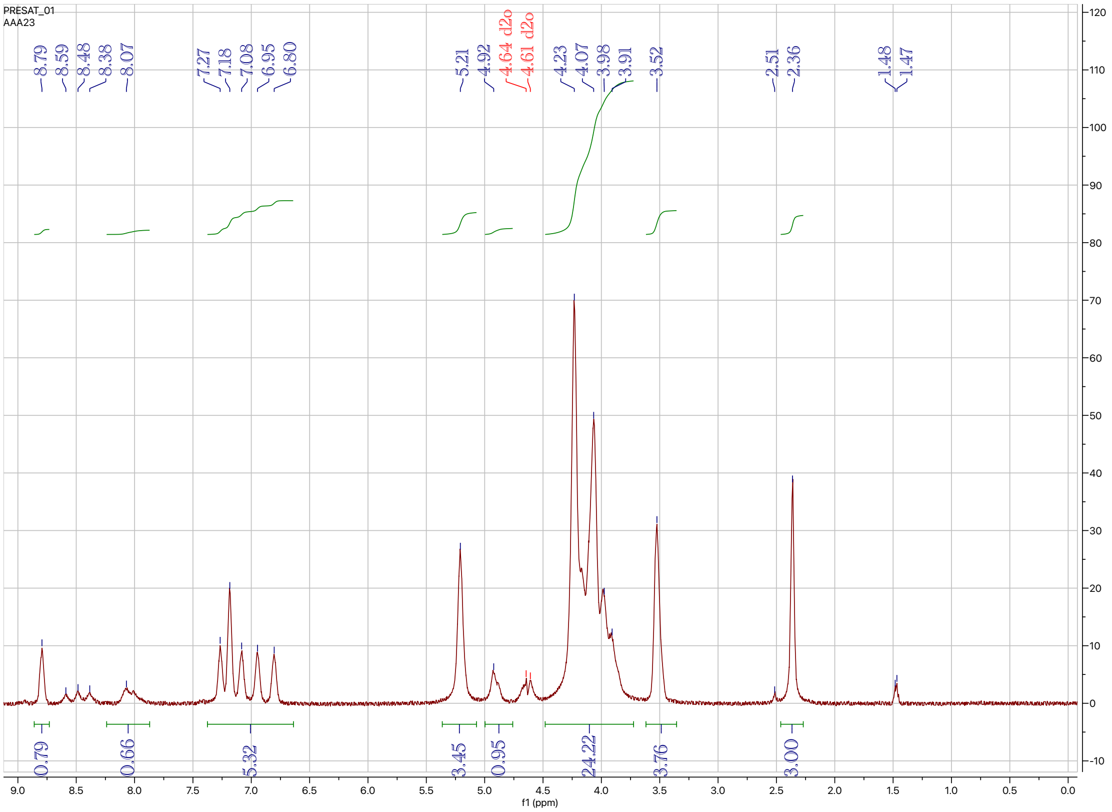

Figure S9. ^{1}H NMR spectra for chitosan derivative NC-2cR1.

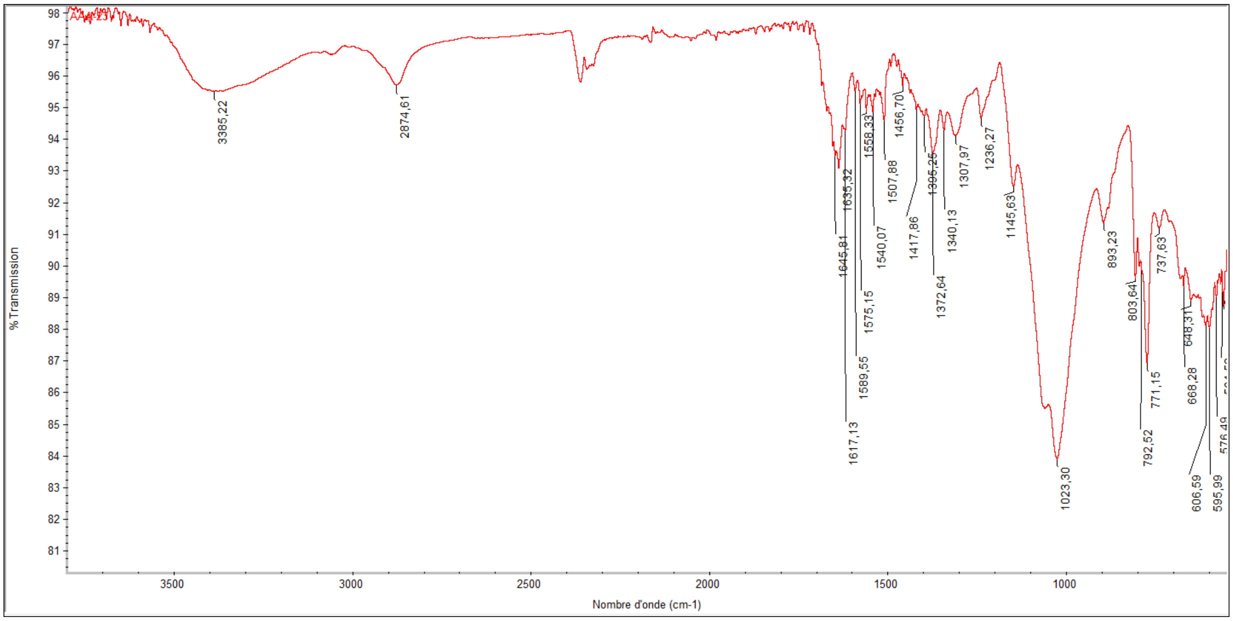

Figure S10. FTIR spectra for chitosan derivative NC-2cR1.

S2.7 Characterization of NC-2cR2

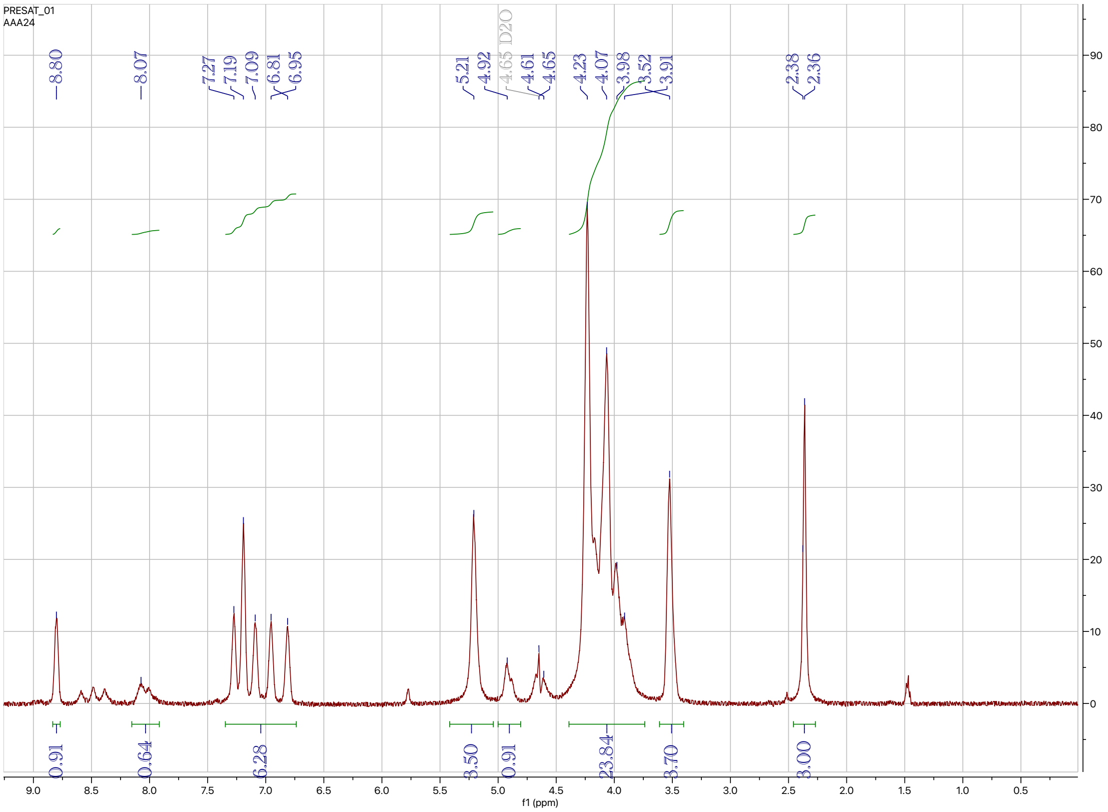

Figure S11. ^1H NMR spectra for chitosan derivative NC-2cR2.

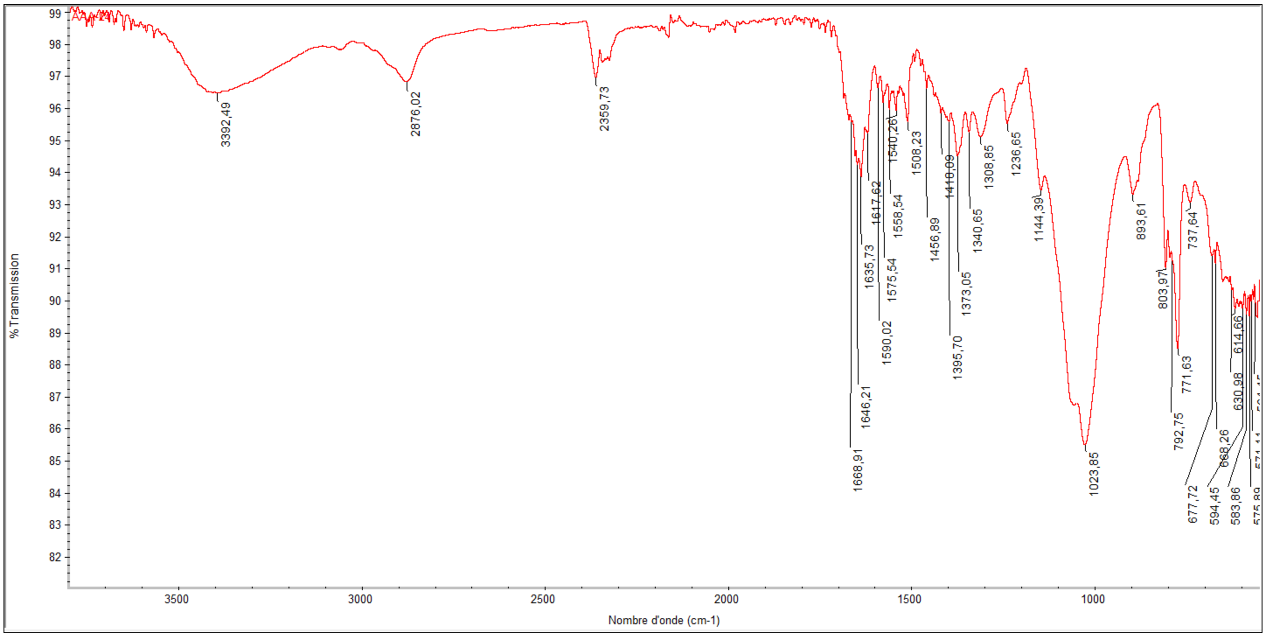

Figure S12. FTIR spectra for chitosan derivative NC-2cR2.

S2.8 Characterization of NC-2d

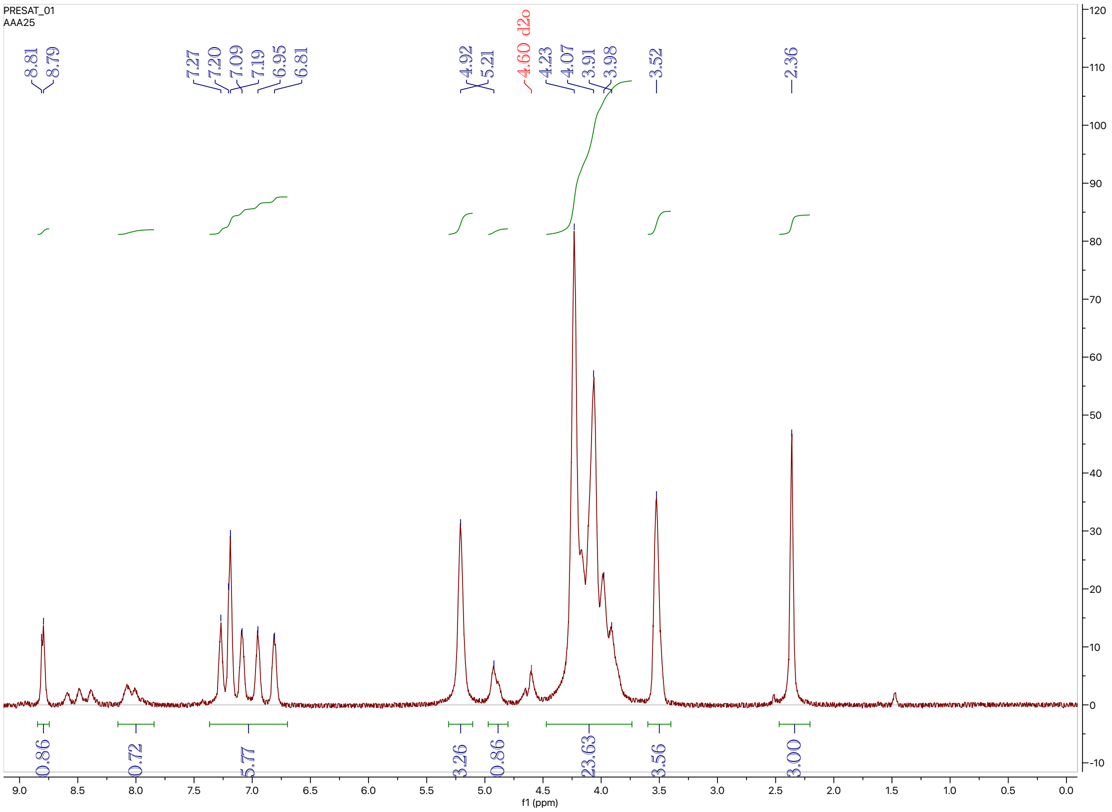

Figure S13. ¹H NMR spectra for chitosan derivative NC-2d.

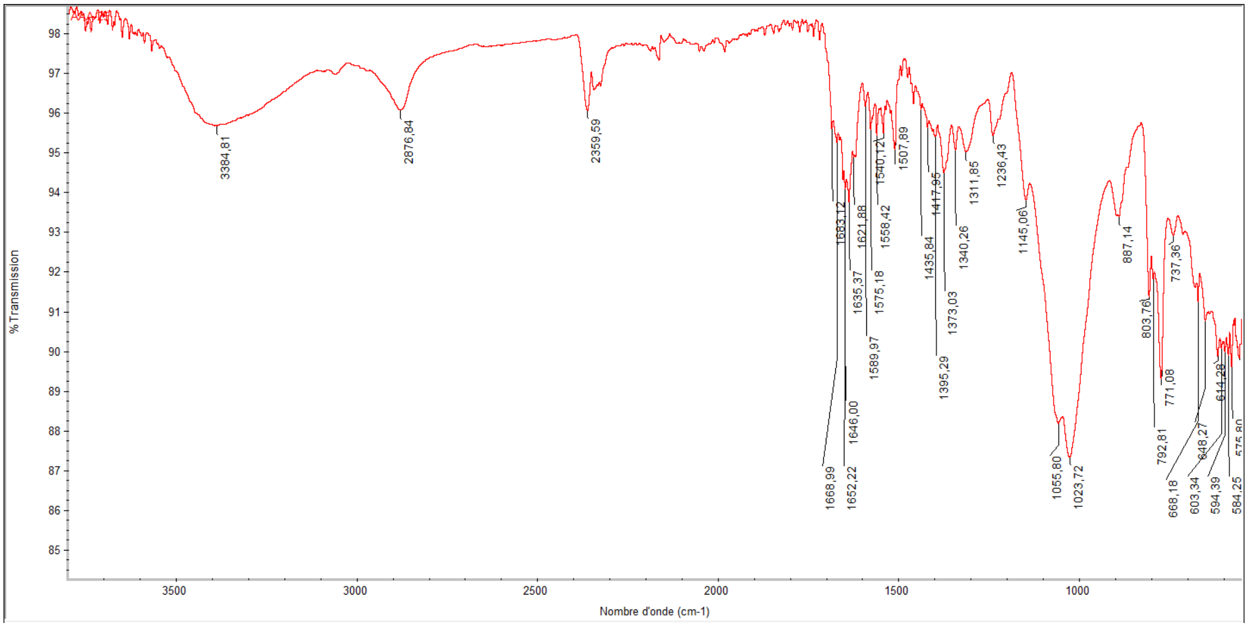

Figure S14. FTIR spectra for chitosan derivative NC-2d.

S2.9 Characterization of NC-2e

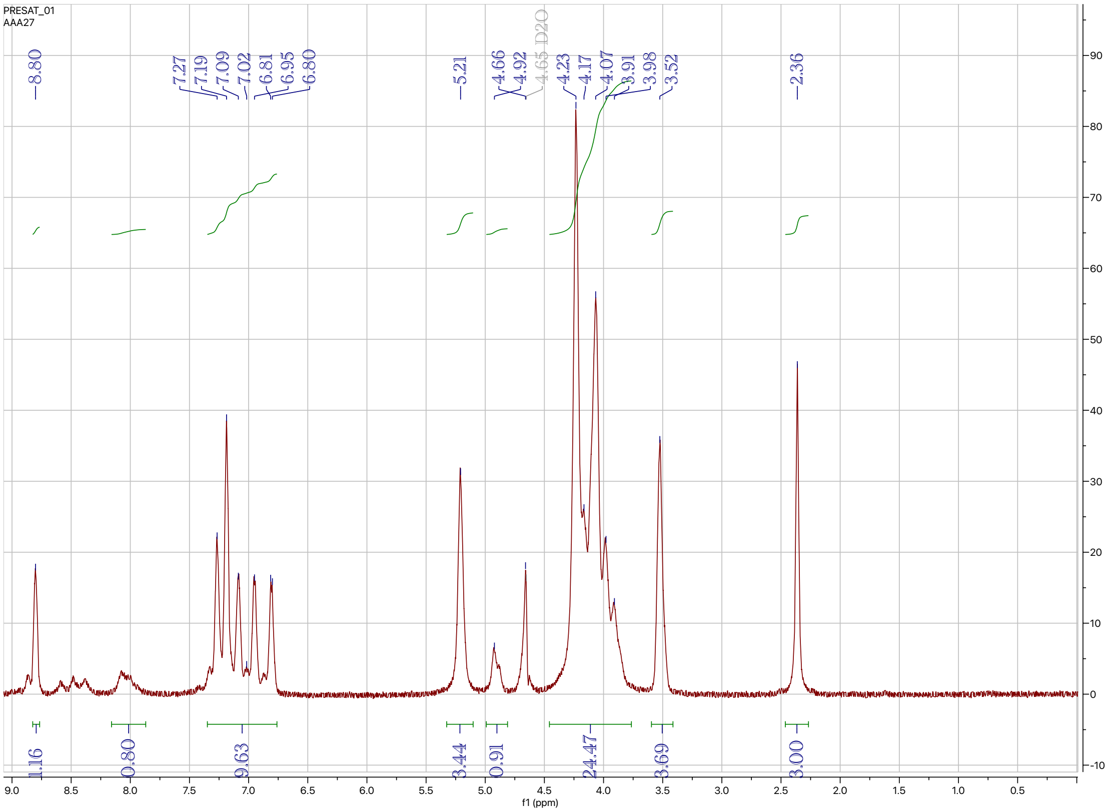

Figure S15. ^{1}H NMR spectra for chitosan derivative NC-2e.

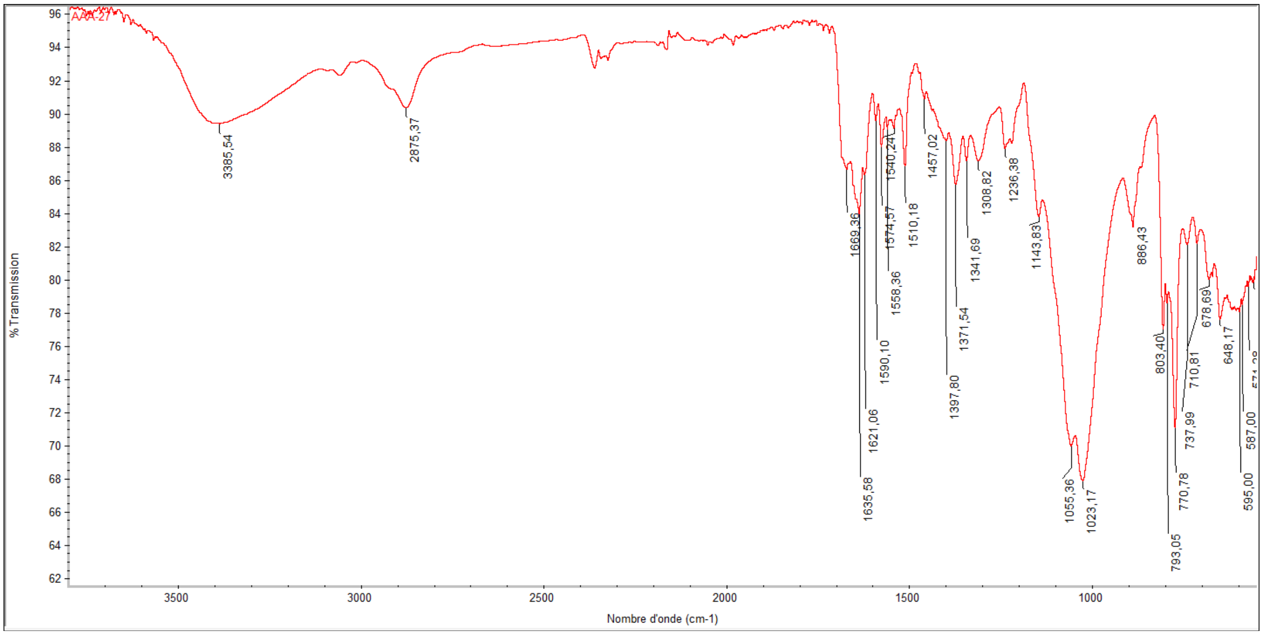

Figure S16. FTIR spectra for chitosan derivative NC-2e.

S2.10 Characterization of NC-3b

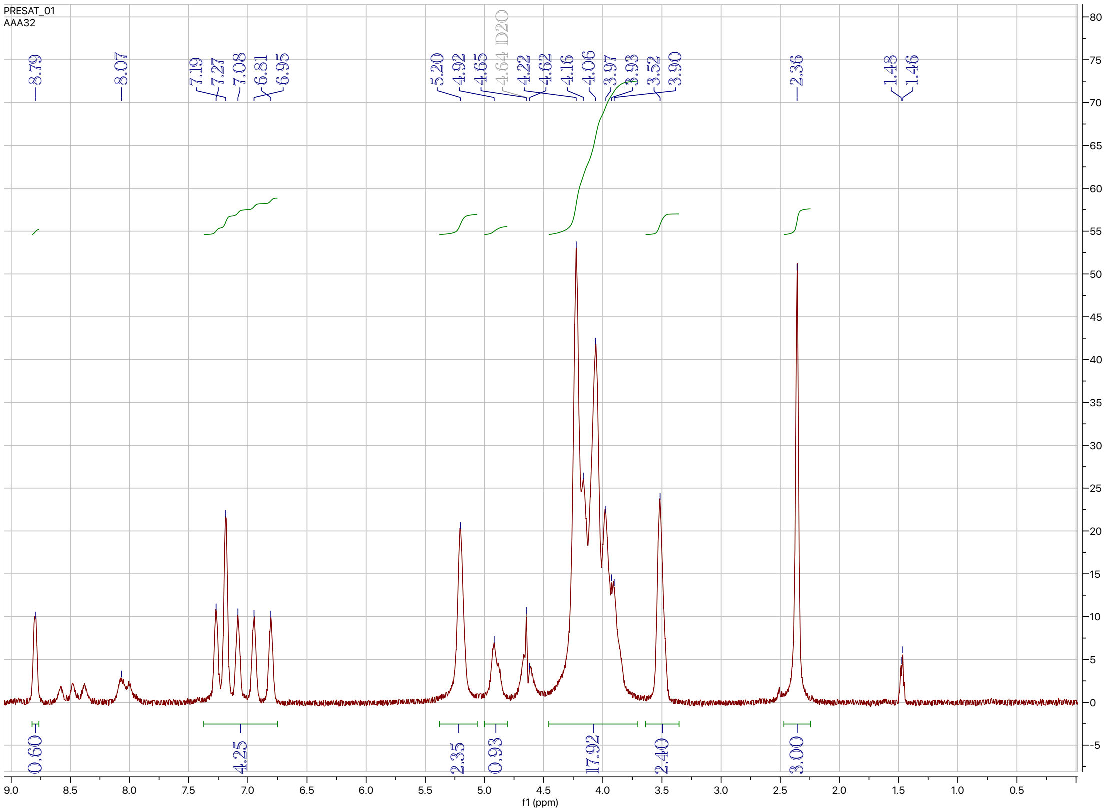

Figure S17. ^{1}H NMR spectra for chitosan derivative NC-3b.

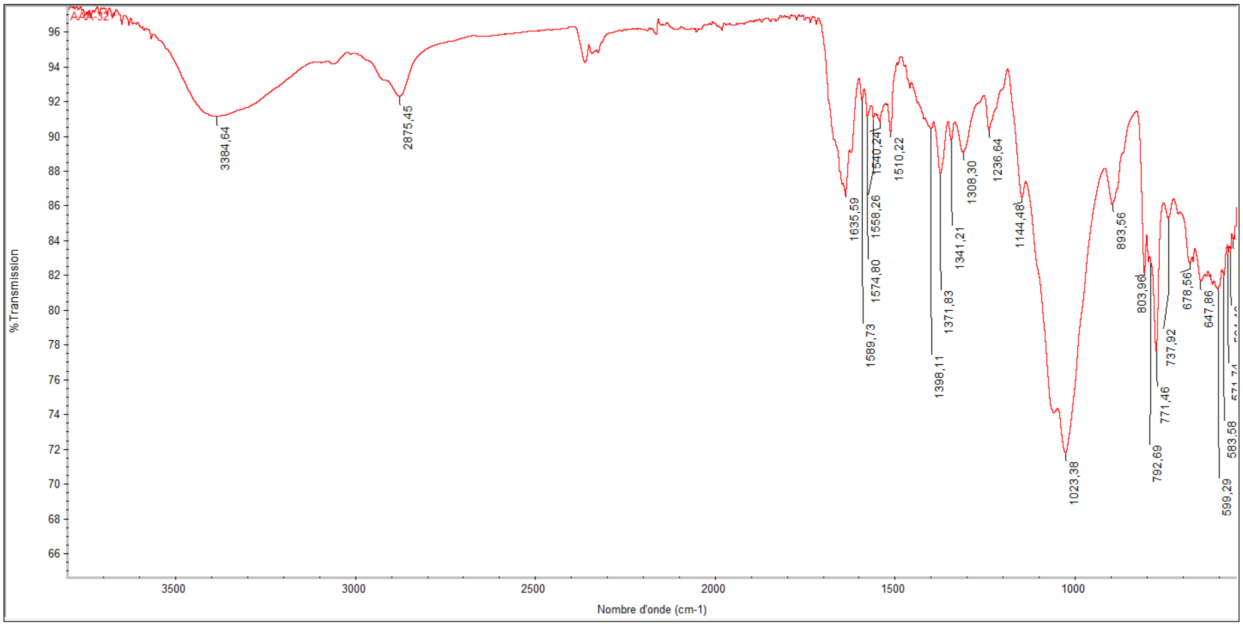

Figure S18. FTIR spectra for chitosan derivative NC-3b.

S2.11 Characterization of NC-3c

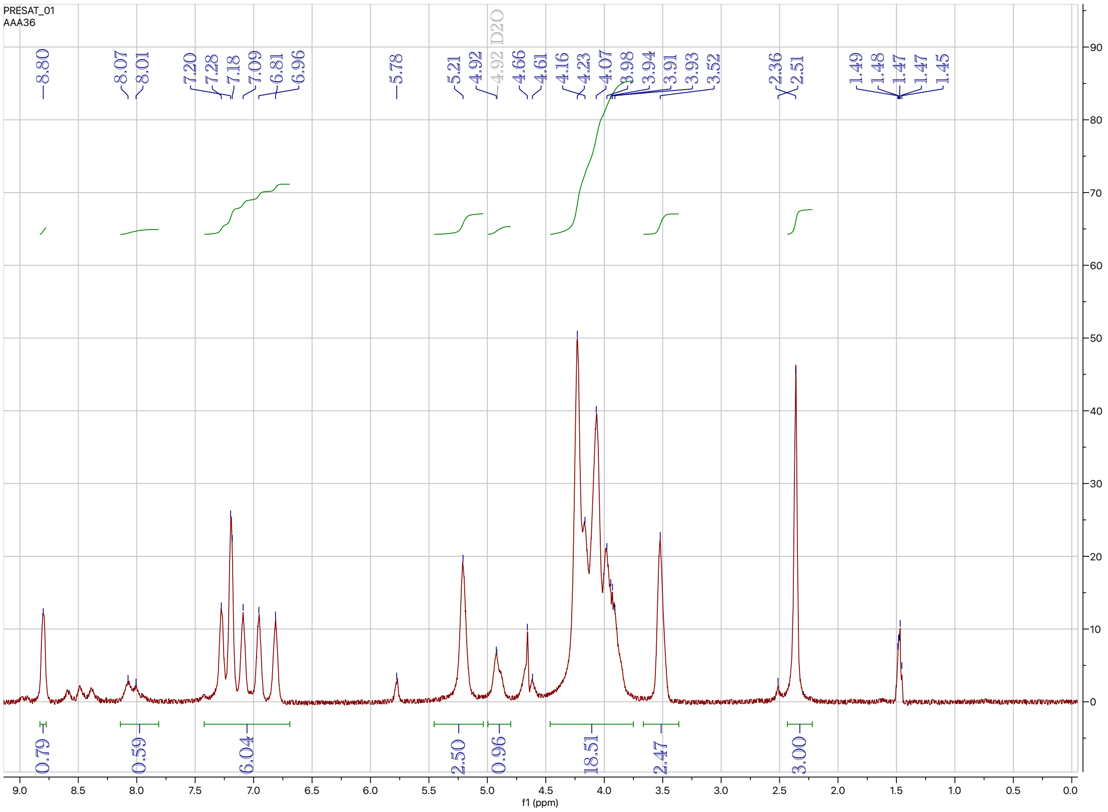

Figure S19. ^{1}H NMR spectra for chitosan derivative NC-3c.

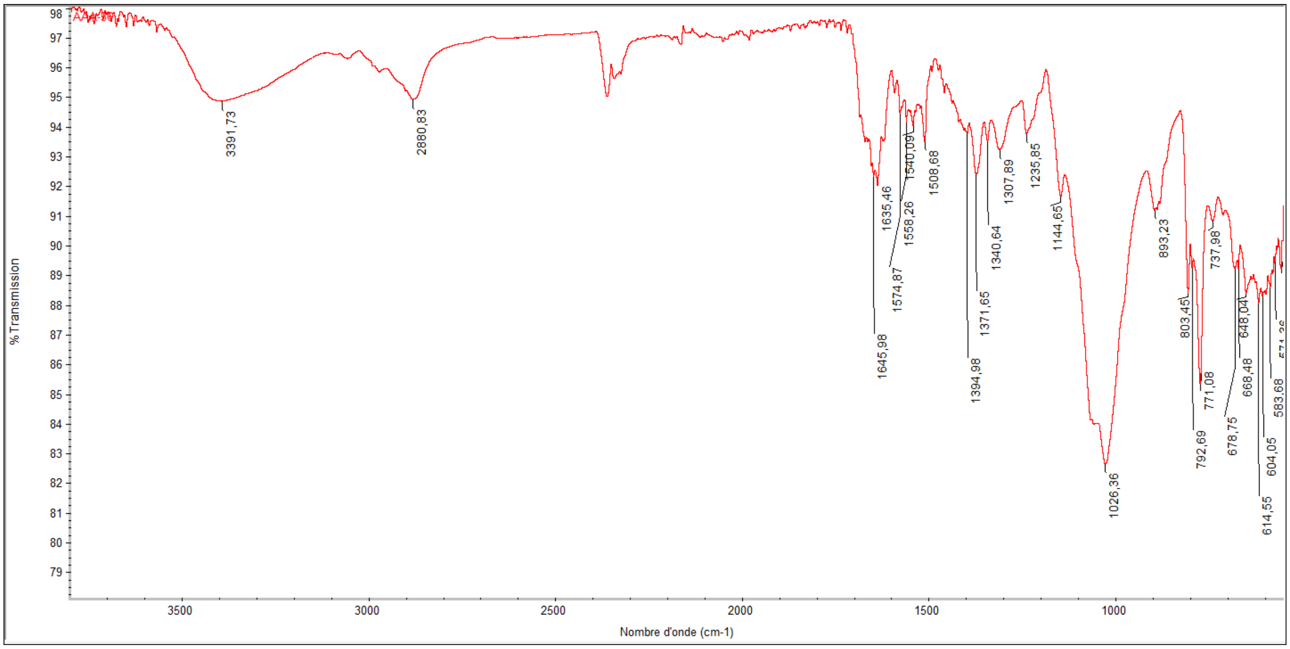

Figure S20. FTIR spectra for chitosan derivative NC-3c.
